# Supplementary material for: The Conformation of Myosin Heads in Relaxed Skeletal Muscle: Implications for Myosin-Based Regulation
Source: Biophys J. 2015 Aug 18;109(4):783–92. doi: 10.1016/j.bpj.2015.06.038 (PMC4547144; doi:10.1016/j.bpj.2015.06.038)
Supplement: Document S1. Supporting Discussion, nine figures, and two tables [file mmc1.pdf]

The conformation of myosin heads in relaxed skeletal muscle: implications for myosin-based regulation

Luca Fusi, Zhe Huang and Malcolm Irving

Randall Division of Cell and Molecular Biophysics, King's College London, London, SE1 1UL (UK)

Supporting Material

Text S1. Integration and Monte Carlo analysis of ME maps

When estimating the integral in spherical coordinates, the 2D-ME intensity distributions calculated at each temperature were multiplied by sinβ. A binary mask was used to select the lobe containing populations RX2 and RX3 (Fig. S8). The boundary of the mask was defined by the contour line of the ME map corresponding to a probability value of 0.045 and by a line running along the local minimum in the intensity between the RX1 and RX2 lobes. Each map was multiplied by the mask and the integral over the selected region was divided by the total over the whole map to calculate $F_{RX(2+3)}$, representing the fraction of heads with RLC regions in the RX2 or RX3 peak (Fig. 4).

In order to evaluate the error on the estimate of $F_{RX(2+3)}$ given the experimental errors on the order parameters, we applied a Monte Carlo simulation in which we calculated the ME maps in five iterations using simulated values of $\langle P_2 \rangle$ and $\langle P_4 \rangle$ for each probe, considered as normal random variables having mean and standard deviation as experimentally determined. The standard deviation (SD) of $F_{RX(2+3)}$ was calculated from the five values obtained in each iteration. This analysis was repeated for data at 2.5, 19.2, 33.0°C in the presence and in the absence of dextran (Fig. S9). The results of this analysis showed that both the relative intensity and position of the ME peaks are not significantly affected by small fluctuations in the order parameters of the four probes. The largest SDs of $F_{RX(2+3)}$ obtained were ±0.025 and ±0.015 for no-dextran and dextran respectively; the errors shown for each data point in Fig. 4 are the pooled standard deviation calculated from dextran and no dextran data.

Table S1. Angular coordinates of the C-lobe BSR-RLCs in the EG frame. θ is the angle between the dipole and the E helix axis, and φ is the angle between the plane defined by the E helix and the dipole and that defined by the E helix and G helix, all in the EG frame (see Fig. S1). L is the distance between the β-carbons of the two residues cross-linked by BSR.

Location	Mutation	L (Å)	θ	φ
**E helix**	**D95CV103C**	**13.1**	**160.2°**	**-172.3°**
**G helix**	**E131CA138C**	**11.1**	**78.3°**	**-1.6°**
**H helix**	**K151CT158C**	**11.4**	**34.9°**	**-175.9°**
**Inter F-G helices**	**T122CK134C**	**11.9**	**61.2°**	**-56.8°**

Table S2. Angular coordinates of relaxed RLC C-lobe orientations. β is the angle between the E-helix and the filament axis and γ is the rotation of the RLC around the E-helix.

	β	γ
**Free RLC (3DTP)**	**158°**	**-60°**
**Blocked RLC (3DTP)**	**131°**	**0°**
**RX1**	**90°**	**-90°**
**RX2**	**165°**	**-70°**
**RX3**	**130°**	**0°**

Supplementary Figures

Fig. S1

A B

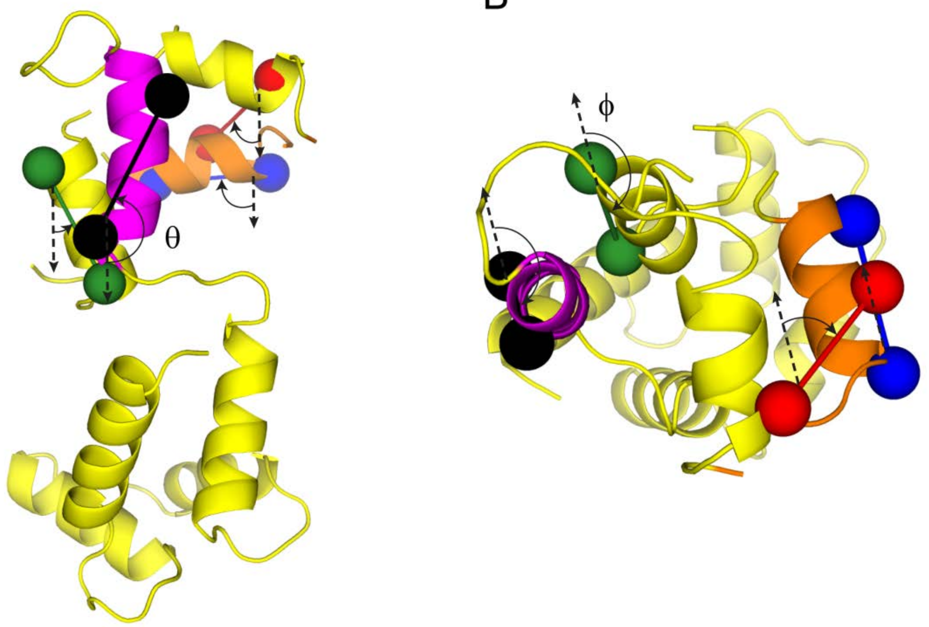

Representation of θ and φ angles of RLC probes in the EG frame. A) θ is the angle between the probe dipole and the vector parallel to the E helix axis (magenta). **B)** View of RLC C-lobe along the E helix axis showing φ, as the angle between the dipole axis and the vector parallel to the G-helix axis (orange). E helix probe, black; G helix probe, blue; H helix probe, green; FG probe, red.

Fig. S2

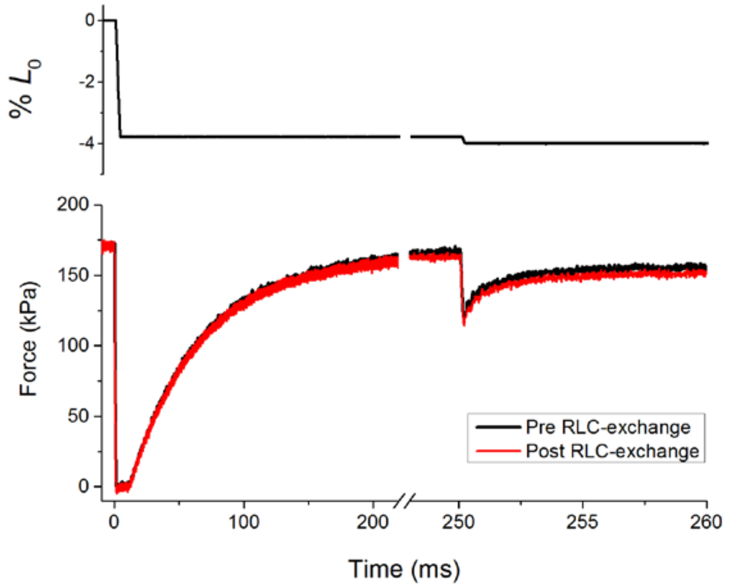

Effect of BSR-RLC exchange on fiber mechanics. Upper panel: imposed fiber length change. Ramp shortening (4% of the initial fiber length L_0 in 3 ms) was applied at the plateau of an isometric contraction; a step release (0.2% L_0 in 0.12 ms) was applied after force redeveloped to the isometric value. Lower panel: force response of the single muscle fiber before (black) and after (red) the BSR-RLC H-helix exchange measured by a capacitance force transducer. The force response to the step is shown on an expanded time scale.

Fig. S3

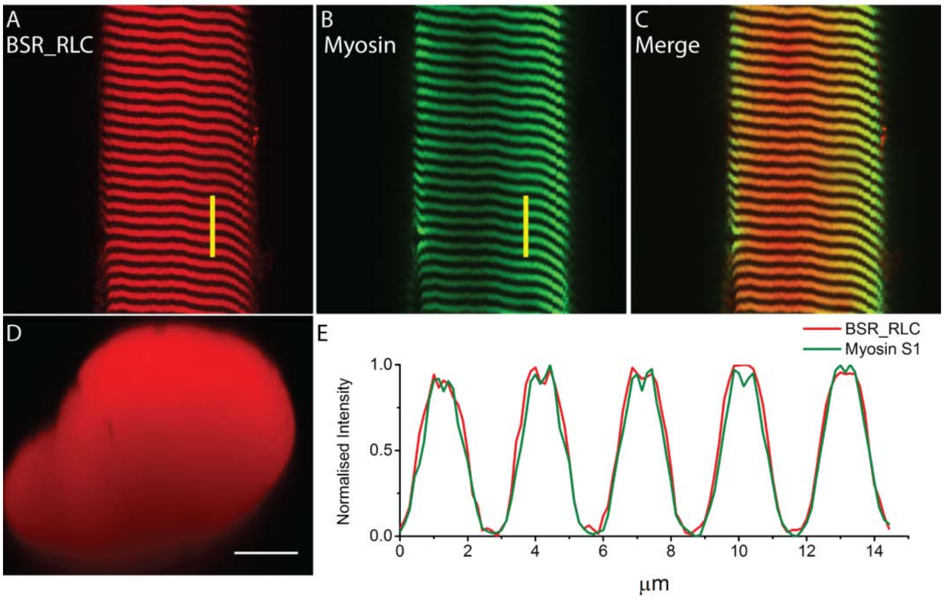

Confocal microscopy of BSR-RLC exchanged fibers. A) Fluorescence of an isolated muscle fiber exchanged with BSR-RLC (E-helix). B) Myosin staining with A4.1025 antibody. C) Merge of the two channels in A and B. D) Orthogonal view showing the fluorescence intensity distribution across the fiber cross-section. Scale bar, 15 µm. E) Normalized intensity profiles along the yellow bar in A and B.

Fig. S4

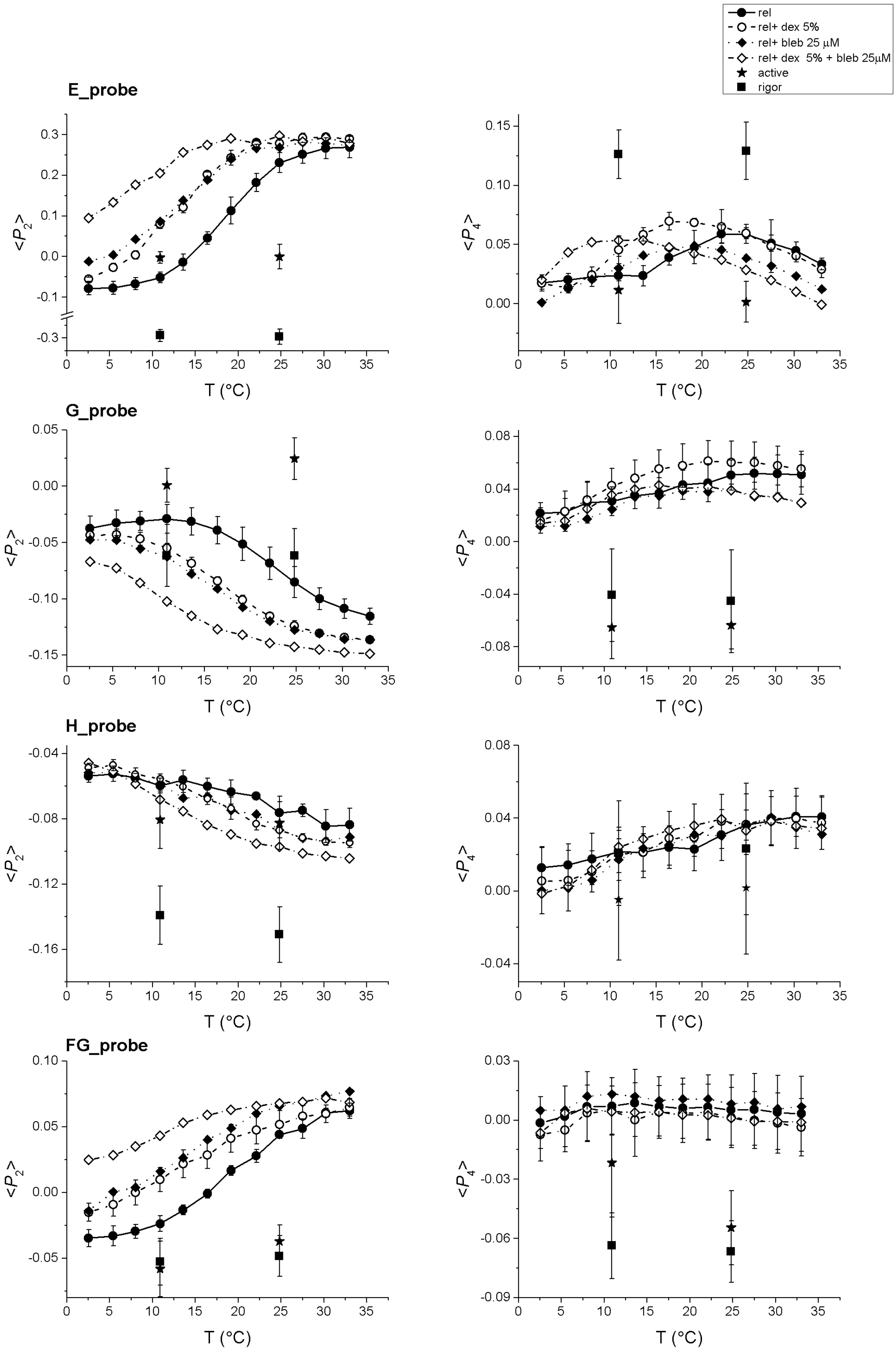

Temperature-dependence of <P_2> and <P_4> of the C-lobe BSR-RLCs. Order parameters <P_2> (left panels, same data as Fig.2 but without error pooling) and <P_4> (right panels)

(mean ± SD) of the four RLC probes in the temperature range 2.5-33.0°C in standard relaxing solution (filled circles and solid line, n=3 fibers), in the presence of 5% dextran T-500 (open circles and dashed line, n=3 fibers) or 25 µM blebbistatin (filled diamonds and dotted line, n=1 fiber), and in the presence of both 5% dextran and 25 µM blebbistatin (open diamonds and dashed/dotted line, n=1 fiber). Order parameters during active contraction and in rigor at 11.0°C and 24.8°C are shown as stars (n=5 fibers) and squares (n=3 fibres) respectively.

Fig. S5

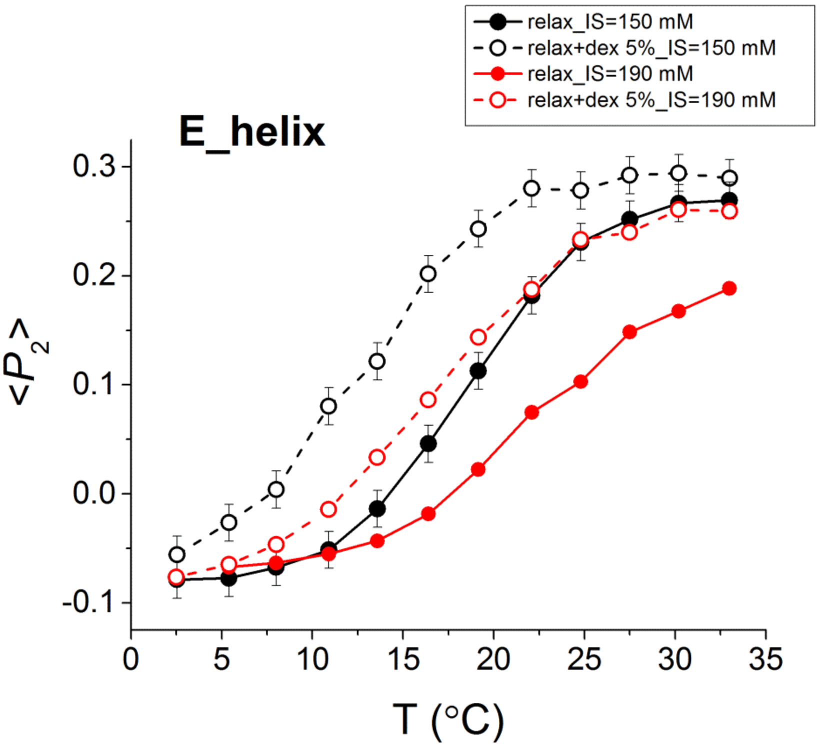

Effect of ionic strength on the orientation of the RLC E-helix probe. Temperature-dependence of the order parameter <P_2> of the E-helix probe in the absence (filled symbols, solid line) and in the presence of 5% dextran T-500 (open symbols, dashed line) in relaxing solution with 150 mM ionic strength and composition given in Table 1 (black symbols, same as in Fig.2), and in relaxing solution with 190 mM ionic strength and with the following composition: TES 100mM, EGTA 25 mM, $MgCl_2$ 7.70 mM, Na_2ATP 5.5 mM, Na_2CP 20 mM, Glutathione 10 mM (red symbols).

Fig. S6

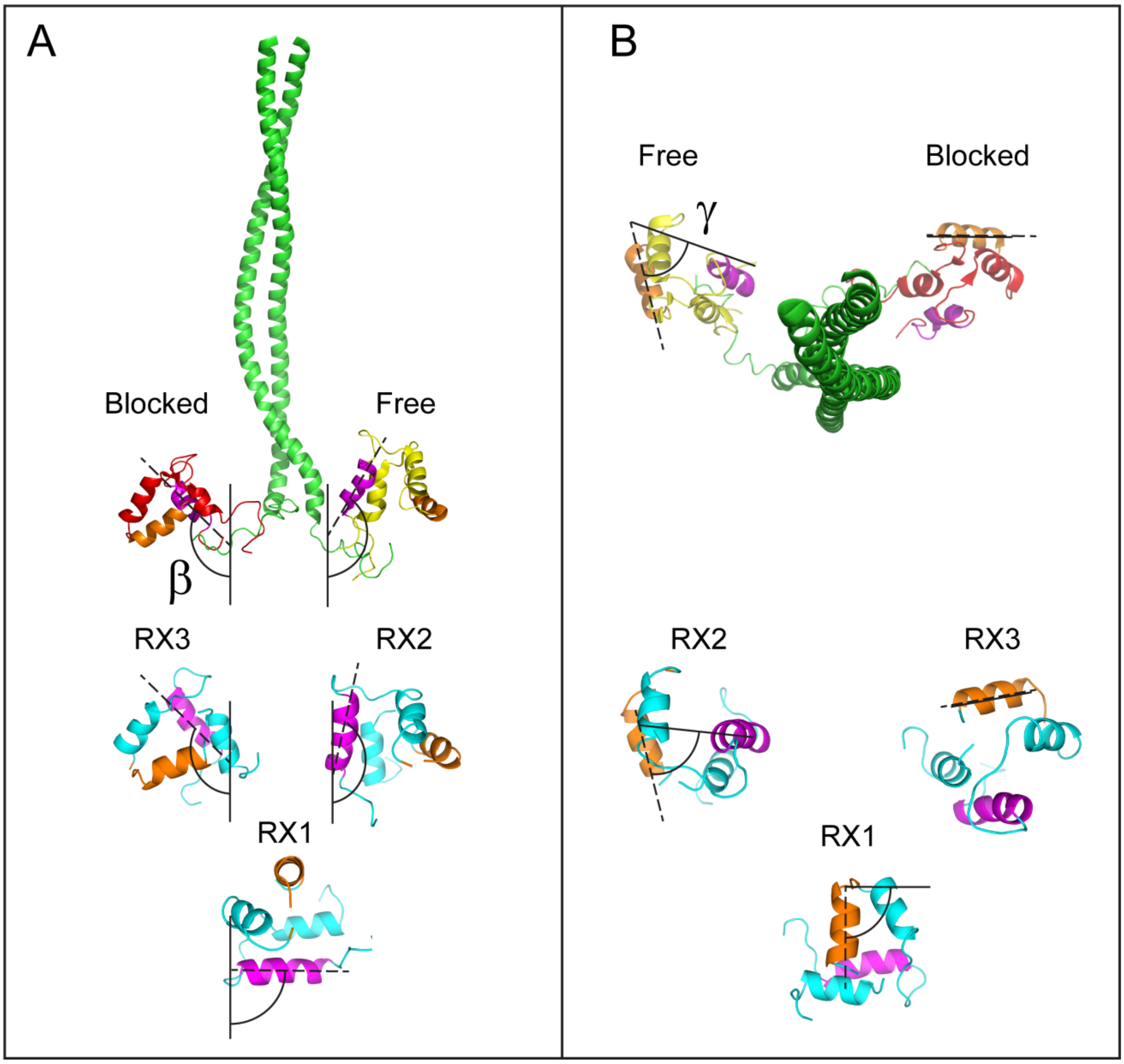

RLC C-lobe orientations in relaxed muscle. A) The angle β (see Table S2) between the E helix axis (magenta, dashed lines) and the filament axis (solid lines) is shown for the RLC C-lobes of the blocked and free heads in the IHM (3DTP), and for the *in situ* orientations RX1, RX2, and RX3. **B)** View along the filament axis showing γ, as the projected angle between the E helix axis (solid lines) and the G helix axis (orange, dashed lines), for each RLC C-lobe orientation.

Fig. S7

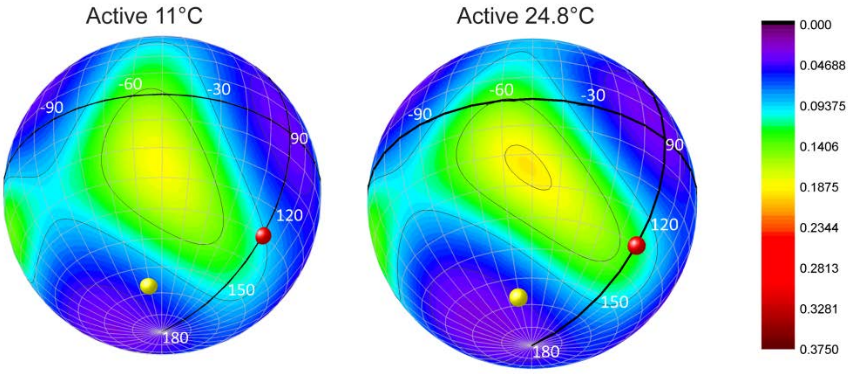

RLC orientation distribution during isometric contraction at 11.0°C and 24.8°C. Spherical plots of maximum entropy (ME) contour maps (as in Fig.3) showing the probability distribution of RLC orientations during active contraction. β=90° at the equator and γ=0°at the meridian. Red and yellow spheres correspond to the RLC orientations in the EG frame calculated for the blocked (β=131°, γ=0°) and free head (β=158°, γ=-60°) in the IHM (Fig. 3F).

Fig. S8

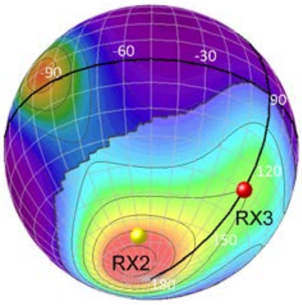

Integration of spherical ME plots. RLC orientation distribution at 24.8°C showing the integrated region of the map containing populations RX2 and RX3 (light colors).

Fig. S9

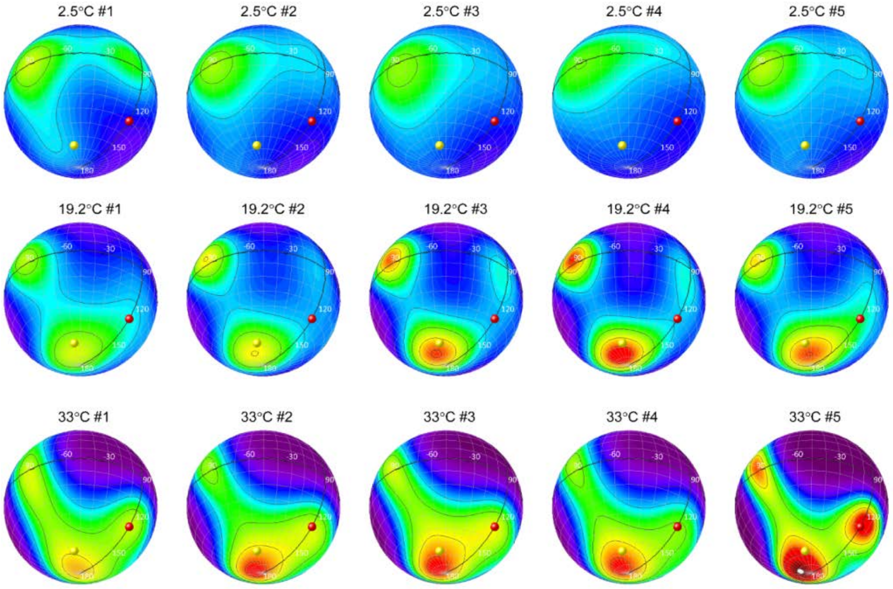

Monte-Carlo simulation of ME plots. RLC orientation distributions obtained in five iterations using simulated values of $<P_2>$ and $<P_4>$ for each probe at three temperatures (2.5, 19.2, 33°C).

Movie S1. Location of BSR probes in the C lobe of RLC. Bifunctional rhodamine (BSR) probes in the C-lobe of the regulatory light chain (RLC, yellow) are represented by rods cross-linking pairs of spheres denoting the inserted cysteines residues. E helix probe, black; G helix probe, blue; H helix probe, green; FG probe, red.

Movie S2. Temperature-dependence of RLC-orientation distribution in relaxing conditions. Frame-sequence of spherical plots of maximum entropy (ME) contour maps (as in Fig.3) showing the probability distribution of RLC orientations in the temperature range 2.5-33.0°C in standard relaxing solution. β=90° at the equator and γ=0°at the meridian. Red and yellow spheres correspond to the RLC orientations in the EG frame calculated for the blocked (β=131°, γ=0°) and free head (β=158°, γ=-60°) in the IHM (Fig. 3F).
