# Supplementary figures and images for: The Conformation of Myosin Heads in Relaxed Skeletal Muscle: Implications for Myosin-Based Regulation
Source: Biophys J. 2015 Aug 18;109(4):783–92. doi: 10.1016/j.bpj.2015.06.038 (PMC4547144; doi:10.1016/j.bpj.2015.06.038)

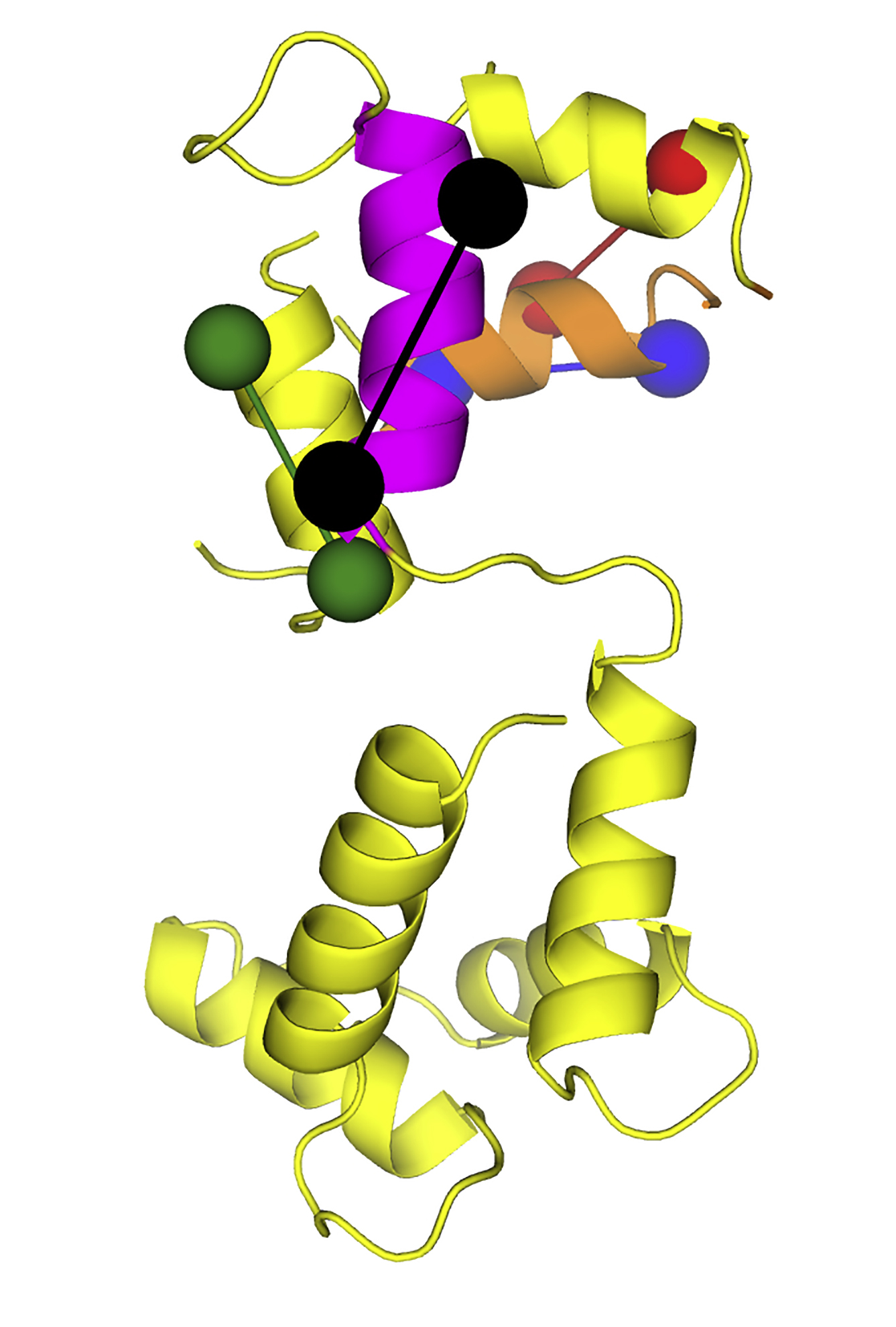

Supplement: Movie S1. Location of BSR probes in the C-lobe of RLC — BSR probes in the C-lobe of the RLC (yellow) are represented by rods cross-linking pairs of spheres denoting the inserted cysteines residues. E helix probe, black; G helix probe, blue; H helix probe, green; FG probe, red. [file mmc2.jpg]

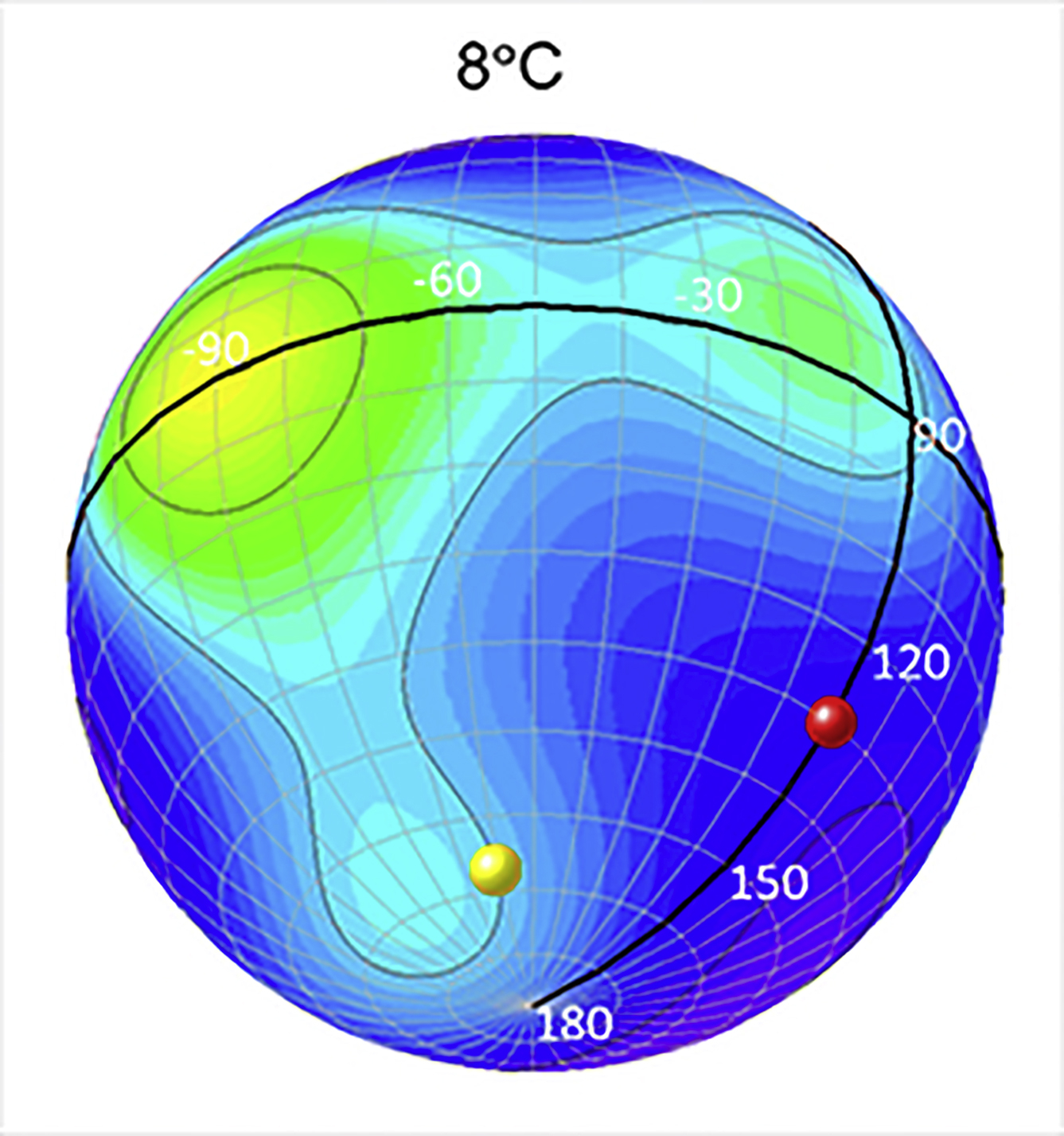

Supplement: Movie S2. Temperature dependence of RLC-orientation distribution in relaxing conditions — Frame sequence of spherical plots of ME contour maps (as in Fig.3) showing the probability distribution of RLC orientations in the temperature range 2.5–33.0°C in standard relaxing solution. β = 90° at the equator and γ = 0°at the meridian. Red and yellow spheres correspond to the RLC orientations in the EG frame calculated for the blocked (β = 131°, γ = 0°) and free head (β = 158°, γ = −60°) in the interacting heads motif (Fig. 3 F). [file mmc3.jpg]
