# Supplementary material for: The Conformation of Myosin Heads in Relaxed Skeletal Muscle: Implications for Myosin-Based Regulation
Source: Biophys J. 2015 Aug 18;109(4):783–92. doi: 10.1016/j.bpj.2015.06.038 (PMC4547144; doi:10.1016/j.bpj.2015.06.038)
Supplement: Document S2. Article plus Supporting Material [file mmc4.pdf]

Article

The Conformation of Myosin Heads in Relaxed Skeletal Muscle: Implications for Myosin-Based Regulation

Luca Fusi,[1,*] Zhe Huang,[1] and Malcolm Irving[1]
[1]Randall Division of Cell and Molecular Biophysics, King's College London, London, United Kingdom

ABSTRACT In isolated thick filaments from many types of muscle, the two head domains of each myosin molecule are folded back against the filament backbone in a conformation called the interacting heads motif (IHM) in which actin interaction is inhibited. This conformation is present in resting skeletal muscle, but it is not known how exit from the IHM state is achieved during muscle activation. Here, we investigated this by measuring the in situ conformation of the light chain domain of the myosin heads in relaxed demembranated fibers from rabbit psoas muscle using fluorescence polarization from bifunctional rhodamine probes at four sites on the C-terminal lobe of the myosin regulatory light chain (RLC). The order parameter $\langle P_2 \rangle$ describing probe orientation with respect to the filament axis had a roughly sigmoidal dependence on temperature in relaxing conditions, with a half-maximal change at ~19°C. Either lattice compression by 5% dextran T500 or addition of 25 μM blebbistatin decreased the transition temperature to ~14°C. Maximum entropy analysis revealed three preferred orientations of the myosin RLC region at 25°C and above, two with its long axis roughly parallel to the filament axis and one roughly perpendicular. The parallel orientations are similar to those of the so-called blocked and free heads in the IHM and are stabilized by either lattice compression or blebbistatin. In relaxed skeletal muscle at near-physiological temperature and myofilament lattice spacing, the majority of the myosin heads have their light chain domains in IHM-like conformations, with a minority in a distinct conformation with their RLC regions roughly perpendicular to the filament axis. None of these three orientation populations were present during active contraction. These results are consistent with a regulatory transition of the thick filament in skeletal muscle associated with a conformational equilibrium of the myosin heads.

INTRODUCTION

Contraction of striated muscle is initiated by calcium-induced changes in the structure of the actin-containing thin filament. Calcium ions released in the cytoplasm following electrical stimulation bind to troponin, triggering the movement of tropomyosin around the filament and uncovering the actin binding sites for the motor or head domains of myosin emerging from the overlapping thick filament (1,2). In skeletal muscle these structural changes in the thin filament are faster than the binding of myosin heads to actin, suggesting that the rate of force generation in physiological conditions may be controlled by the kinetics of structural changes in the thick filament (3,4). Thick filament-based regulatory mechanisms are best characterized in smooth muscle (5), where they replace the troponin/tropomyosin system as the primary regulatory system, and they have received much less attention in skeletal muscle.

Early x-ray diffraction studies of skeletal muscle suggested that the myosin heads in resting muscle are arranged in a quasihelical array near the surface of the thick filaments, and that this helical arrangement is lost on activation (6).

Submitted February 20, 2015, and accepted for publication June 17, 2015.

*Correspondence: luca.fusi@kcl.ac.uk

This is an open access article under the CC BY license (http://creativecommons.org/licenses/by/4.0/).

Editor: E. Ostap.

© 2015 The Authors

0006-3495/15/08/0783/10

Subsequent electron microscopy (EM) studies of isolated thick filaments from invertebrate skeletal muscle identified an asymmetric arrangement of the two heads of each myosin molecule folded back against the myosin tails in the filament backbone (7,8). This conformation, called the J motif or interacting heads motif (IHM), inhibits the ATPase activity of intrinsically regulated myosin filaments (i.e., in muscles lacking the troponin/tropomyosin system) and is associated with the relaxed or OFF state of smooth muscle myosin (9). The IHM has also been observed in the C-zone of cardiac thick filaments, the region containing myosin-binding protein C (MyBP-C) (10,11), and in isolated molecules of vertebrate muscle myosin (12). No high-resolution structures are available for isolated thick filaments from vertebrate skeletal muscle, but x-ray interference studies are consistent with the presence of the IHM in resting intact muscle (3). Functionally, such a structurally inhibited state of myosin in resting skeletal muscle could explain the slow ATP turnover observed in relaxed demembranated fibers from mammalian muscle, which has been called a super-relaxed state (SRX) (13,14). However, the interaction between this potential regulatory structural switch in the thick filament and the well-known regulatory switch in the thin filaments remains obscure.

To better understand the role of myosin head conformations in the concept of thick filament regulation summarized above, we measured the orientation of the regulatory light

http://dx.doi.org/10.1016/j.bpj.2015.06.038

CrossMark

chain (RLC) regions of the myosin heads in the native sarcomeres of demembranated fibers from mammalian muscle. The structural relationship between the thick and thin filaments is preserved in this preparation, and RLC orientation can be measured using fluorescence polarization from bifunctional sulforhodamine (BSR) probes at four different sites on the C-terminal lobe of the RLC. X-ray diffraction studies on this preparation showed that the quasihelical organization of myosin heads on the thick filament surface in relaxing conditions depends strongly on temperature (15–18). We therefore used this protocol as a model system to investigate the transition between conformations of the myosin heads with different degrees of helical order, overcoming limitations of a previous fluorescence polarization study by using a skeletal muscle isoform of RLC rather than a smooth muscle isoform (19), and a milder exchange protocol to introduce labeled RLCs into muscle fibers. To further constrain the interpretation, we also investigated how the relationship between myosin head conformation and temperature depends on the lateral spacing between the thick and thin filaments, and on inhibition of myosin by blebbistatin (20).

MATERIALS AND METHODS

Muscle fiber preparation

~6 mm long segments of demembranated fibers were dissected from rabbit psoas muscle as previously described (21). Fibers were mounted in the experimental trough between a force transducer and a loudspeaker motor, and the extremities were fixed using 5% glutaraldehyde in rigor solution and glued to the clips with shellac dissolved in ethanol. The average sarcomere length was 2.42 ± 0.05 μm and the average cross-sectional area was 4542 ± 1186 μm^2 (mean ± SD, $n = 12$ fibers). The temperature of the trough was controlled with ±0.1°C accuracy using Peltier modules under the feedback control of a driver unit (Electrondynamics, Southampton, UK). The solution compositions (see Table 1) were calculated using software kindly provided by Prof. Earl Homsher; 10 mM DTT was added to all solutions and 1 mg/ml creatine phosphokinase (C3755, Sigma, Gillingham, UK) was added to relaxing, preactivating and activating solutions. The ionic strength of each solution was 150 mM except where stated and the pH was 7.1 at 11°C.

Preparation of BSR-RLCs

Four double-cysteine mutants of chicken skeletal RLC (D95C/V103C (E helix), E131C/A138C (G helix), K151C/T158C (H helix), and T122C/K134C (linking F and G helices)) were obtained by site-directed mutagenesis, expressed in *Escherichia coli*, and purified as previously described (19,22) (Fig. S1 in the Supporting Material). The two native cysteines at position 125 and 154 were replaced by alanine to avoid nonspecific labeling. Each pair of introduced cysteines was cross-linked with BSR (B10621, Invitrogen, Waltham, MA) to give 1:1 BSR-RLC conjugates that were purified by reverse-phase high-performance liquid chromatography to >95% homogeneity. Specificity and stoichiometry of BSR labeling were determined by reverse-phase high-performance liquid chromatography and mass spectrometry.

Exchange of BSR-RLC mutants into muscle fibers

Muscle fibers were first transferred to rigor solution at ~1°C, and then RLC-extracting solution (20 mM EDTA, 50 mM KPr, 10 mM potassium phosphate buffer, pH 7.1) at 1°C for 3′, and finally to RLC-extracting solution containing 0.5 mg/ml (~25 μM) BSR-RLC at 19°C for 30′. Fibers were washed in relaxing solution and then incubated at 10°C for 20′ in relaxing solution containing 0.5 mg/ml of rabbit skeletal muscle troponin complex (Tn, Life Diagnostics, West Chester, PA) and for a further 20′ in relaxing solution containing 0.5 mg/ml of wild-type chicken skeletal muscle troponin C (TnC), expressed in *E. coli*, and purified as previously described (23), to replace Tn/TnC lost during the RLC exchange. Before and after the exchange protocol the fiber was activated twice at 11°C by temperature-jump (21). A ramp-release (4% of the initial fiber length (L_0) complete in 3 ms) and a step-release (0.2% L_0 complete in 0.12 ms) after the force redevelopment following unloaded shortening were applied at the plateau of the isometric contraction to check the mechanical performance of the RLC-exchanged fiber (Fig. S2). The concentration of BSR-RLC in the fiber ($C_{BSR\text{-}RLC}$), calculated from fluorescence intensity (4), was 39 ± 10 μM (mean ± SD, $n = 6$ fibers) and therefore the exchanged fraction was 26% assuming that the concentration of native RLC in demembranated fibers from rabbit psoas muscle is 150 μM (24). Maximum isometric force was 224 ± 23 kPa at 11°C and force recovery after RLC exchange was 95 ± 4% (mean ± SD, $n = 12$ fibers). Samples for confocal microscopy (Fig. S3) were prepared by stretching the relaxed fiber to a sarcomere length of 3.0 μm, and then fixing in 4% formaldehyde at room temperature for 5′. The fiber was then incubated in antimyosin antibody (A4.1025, kindly provided by Prof. Mathias Gautel) for 1 h at room temperature and in secondary antibody labeled with Alexa Fluor 488 for 1 h at room temperature, and finally transferred to a microscope slide in a mounting medium. Images were acquired using a Zeiss LSM 510 Meta (Zeiss, Jena, Germany).

Experimental protocol and data analysis

The temperature of the experimental trough was increased in steps of 3°C in the range 2.5–33.0°C, and the polarized fluorescence intensities from the four BSR-RLCs were recorded in relaxing conditions at each temperature using a custom-built setup (4) and used to calculate the second- and fourth-rank order parameters of the orientation distribution of the BSR dipole, $\langle P_2 \rangle$ and $\langle P_4 \rangle$, respectively (25). The order parameter $\langle P_{2d} \rangle$ quantifying rapid probe motion was calculated from the polarized intensities recorded with *X*- and *Y*- illumination (in line with and perpendicular to the measured fluorescence emission, respectively (4)). In relaxing conditions $\langle P_{2d} \rangle$ was 0.867 ± 0.004 for the E helix probe, 0.849 ± 0.018 for the G

TABLE 1 Composition of the Physiological Solutions

	Imidazole	EGTA	Ca-EGTA	HDTA	KPr	$MgAc_2$	Na_2ATP	Na_2CP	pCa
Relaxing	25	10	–	–	11.8	6.89	5.56	20	9
Preactivating	25	0.1	–	9.9	11.7	6.48	5.56	20	9
Activating	25	–	10	–	11.7	6.39	5.65	20	4.7
Rigor	25	10	–	–	100.3	1.50	–	–	9

All the concentrations are in mM. EGTA; HDTA, 1,6 diaminohexane-*N*,*N*,*N*′,*N*′-tetraacetic acid; KPr, potassium propionate; $MgAc_2$, magnesium acetate; ATP; Na_2CP, phosphocreatine disodium salt; pCa = $-\log[Ca^{2+}]$. Mg^{2+}-ATP concentration is 5 mM and free Mg^{2+} is 1 mM.

helix probe, 0.760 ± 0.009 for H helix probe, and 0.827 ± 0.017 for the FG probe (mean ± S.D., $n = 3$ fibers), indicating that the amplitude of such motion is small. $\langle P_{2d} \rangle$ was not sensitive to temperature in the range studied here. The pH of the solutions was lower at higher temperature, but order parameters measured at 25°C in a relaxing solution with pH set to 7.1 at 11°C were not significantly different from those with pH set to 7.1 at 25°C, indicating that the probe orientation in relaxing solution is not sensitive to temperature-induced changes in pH. The effect of dextran on the temperature dependence of $\langle P_2 \rangle$ and $\langle P_4 \rangle$ in relaxing conditions was investigated in three independent experiments for each RLC probe (n =12 fibers) (see Fig. 2). The effect of blebbistatin in the presence and in the absence of dextran on the relaxed values of $\langle P_2 \rangle$ and $\langle P_4 \rangle$ was tested in one experiment for each mutant ($n = 4$). The errors shown for relaxing conditions (see Fig. 2) are pooled standard deviations for all the data for each probe in the presence and absence of dextran; the original data for each temperature and condition without error pooling are shown in Fig. S4.

To describe the in situ RLC-orientation, we defined a reference frame (the EG frame (26)) using the crystallographic structure of nucleotide-free myosin S1 from chicken skeletal muscle (see Fig. 1 (27)). The orientation of the RLC domain is described by β, the angle between the E helix (see Fig. 1, *magenta*) and the thick-filament axis, and γ, the twist angle around the E helix. $\gamma = 0$ when the G helix (see Fig. 1, *orange*) is in the plane defined by the E helix and the thick-filament axis, and $\gamma > 0$ for a counterclockwise rotation of the lobe viewed from the + end of the E helix. The orientation of each BSR probe in this frame was described by two angles calculated from the coordinates of the β carbons of the two attachment points, assuming that the dipole axis is parallel to the line joining those points. θ is the angle between the dipole and the E helix, and φ is the angle between the plane defined by the E helix and the dipole and that defined by the E and G helices (Table S1; Fig. S1). These angular coordinates and the measured order parameters for the four probes were used in a maximum entropy (ME) algorithm (28,29) to calculate the smoothest distribution of RLC-orientations consistent with the data. The two-dimensional contour map denoting the probability of each (β,γ) orientation (with $0° < \beta < 180°$ and $0° < \gamma < 360°$) was projected onto the surface of a sphere using Origin software (OriginLab, Northampton, MA), in which β is the polar angle (latitude) and γ the azimuthal angle (longitude). This representation has the advantage of avoiding distortion of the distribution introduced in a Cartesian representation and of showing only one of the two equivalent solutions (β,γ) and ($180°-\beta$, $180°+\gamma$) associated with the dipole ambiguity and the antiparallel arrangement of myosin motors in the two halves of the thick filament (30). The methods used for integration of specific regions of the ME maps are discussed in the Supporting Material.

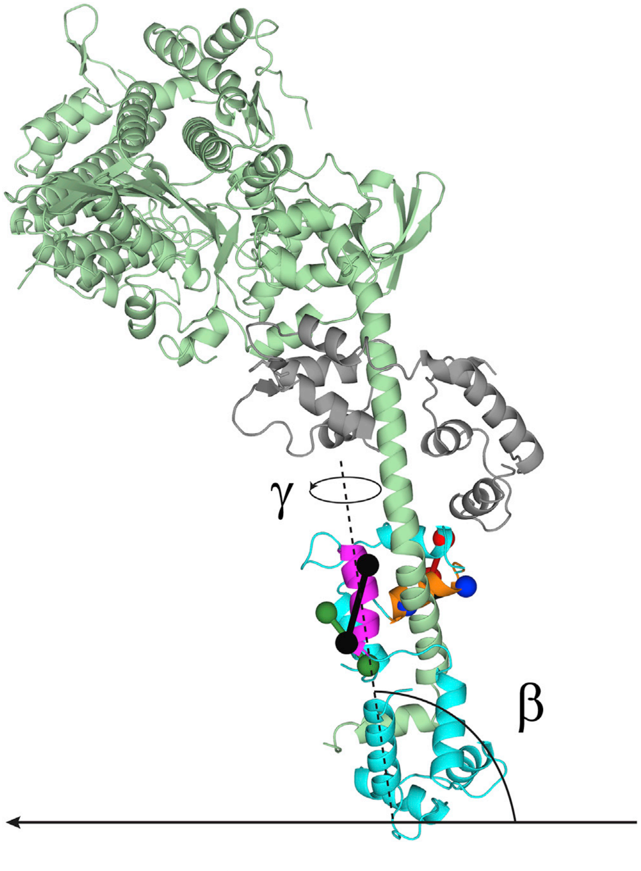

FIGURE 1 Locations of BSR probes on the myosin head domain. BSR probes in the C-lobe of the RLC (*cyan*) are represented by rods cross-linking pairs of spheres denoting the inserted cysteine residues. E helix probe, black; G helix probe, blue; H helix probe, green; FG probe, red. The orientation of the RLC region is described using angular coordinates defined by helices E and G (*magenta* and *orange*, respectively): β is the angle between the E helix axis (*dashed line*) and the thick filament axis (*solid line*), and γ is the twist angle around the E helix axis. Myosin heavy chain, pale green; essential light chain, gray. Coordinates for nucleotide-free chicken skeletal myosin (27). This figure was generated using the PyMOL Molecular Graphic System (DeLano Scientific, LLC, Palo Alto, CA).

RESULTS

Temperature dependence of the order parameters of RLC probes in relaxed muscle fibers

Mutants of chicken skeletal RLC, with native cysteines replaced by alanine and new cysteine pairs introduced in its C-lobe at positions 95–103 (E helix), 131–138 (G helix), 151–158 (H helix), or 122–134 (in the F and G helices, respectively), were labeled by cross-linking cysteine pairs with BSR (Fig. 1; Fig. S1; Movie S1). The E helix probe was designed with its dipole axis approximately parallel to the myosin lever arm to be sensitive to lever arm tilting. Each BSR-RLC was exchanged into permeabilized fibers from rabbit psoas muscle using a mild exchange protocol to better preserve the native structure and function of the thick filaments (see Materials and Methods section). This protocol led to replacement of ~26% of the native RLC by BSR-RLC, which was uniformly distributed along the A-band of the sarcomere and across the fiber width (Fig. S3). Moreover, the function of myosin motors was not affected by RLC exchange: isometric force recovery after exchange was >95%, and both the rate of force redevelopment following unloaded shortening and the force transient elicited by a 0.2% step release in fiber length were similar to those before exchange (Fig. S2).

The polarized fluorescence from the four RLC probes was measured in the temperature range 2.5–33.0°C in relaxing conditions at maximum myofilament overlap (sarcomere length 2.40 μm) and used to calculate the second- and fourth-rank order parameters of the orientation distribution of each BSR-RLC in the fiber, $\langle P_2 \rangle$ and $\langle P_4 \rangle$, respectively. $\langle P_2 \rangle$ is a measure of how parallel the probe dipole is to the filament axis, and varies from +1 for parallel to −1

for perpendicular. $\langle P_4 \rangle$ is a higher-order harmonic term, giving higher resolution angular information (25).

Increasing the temperature in relaxing solution induced reproducible changes in $\langle P_2 \rangle$ for each probe (Fig. 2, *solid line* and *solid circles*), with smaller changes in $\langle P_4 \rangle$ (Fig. S4). The temperature dependence of $\langle P_2 \rangle$ for each probe had a roughly sigmoidal shape with a transition temperature (the temperature at which the change in $\langle P_2 \rangle$ is half maximal) of ~19°C. The largest change in orientation was observed for the E helix, with $\langle P_2 \rangle$ becoming more positive at high temperature, indicating that the E helix became more parallel to the filament axis. The FG probe also became more parallel to the filament axis ($\langle P_2 \rangle$ became more positive), but the G and H helices became more perpendicular ($\langle P_2 \rangle$ became more negative).

The effect of myofilament lattice spacing on RLC orientation was investigated by adding 5% dextran T500 to the relaxing solution, which is expected to restore the filament lattice spacing to that of the intact muscle (31). For all four probes the main effect of osmotic compression was to shift the $\langle P_2 \rangle$-T relations to lower temperatures, with a decrease of the transition temperature by ~5°C (Fig. 2, *dashed lines* and *open circles*). At high temperature $\langle P_2 \rangle$ for the E helix was slightly but consistently higher in the presence of dextran, and that for the G and H helices was slightly lower. The temperature dependence of $\langle P_2 \rangle$ was also affected by the ionic strength of the relaxing solution: the transition temperature was ~4°C lower at 150 mM ionic strength than at 190 mM (Fig. S5).

Blebbistatin, a myosin inhibitor that has been reported to induce ordering of the thick filaments in relaxed muscle fibers (32), also shifted the $\langle P_2 \rangle$-T relations of the four probes in relaxing solution (Fig. 2, *dotted line* and *solid diamonds*). 25 μM blebbistatin displaced the $\langle P_2 \rangle$-T relations to ~5°C lower temperature, similar to the effect of osmotic compression by 5% dextran. The effects of blebbistatin and dextran were additive; in the presence of both 25 μM blebbistatin and 5% dextran T500 the transition temperature was reduced by ~10°C (Fig. 2, *dot-dashed line* and *open diamonds*).

RLC-orientation distribution in relaxed muscle fibers

The orientation of RLC with respect to the filament or fiber axis was described by two angular coordinates (β,γ) using a reference coordinate frame defined by the E and G helices (*magenta* and *orange helices*, respectively, in Fig. 1). β is the angle between the E helix and the filament axis, whereas γ is the rotation of the RLC around the E helix using the G helix to define $\gamma = 0$ (Figs. 1 and S6). The in situ orientation distribution of the RLC in this EG frame was calculated using a ME algorithm (28), a model-free method that determines the smoothest probability distribution of orientations that reproduces the experimental $\langle P_2 \rangle$ and $\langle P_4 \rangle$ values for each probe (Figs. 2 and S4), given the angular coordinates of the probe dipoles in the EG frame (Table S1). These probability distributions were plotted on a sphere (Fig. 3), where β is the latitude and γ is the longitude (β = 90° at the equator and γ = 0° at the meridian), as contour maps with hotter colors denoting more likely orientations.

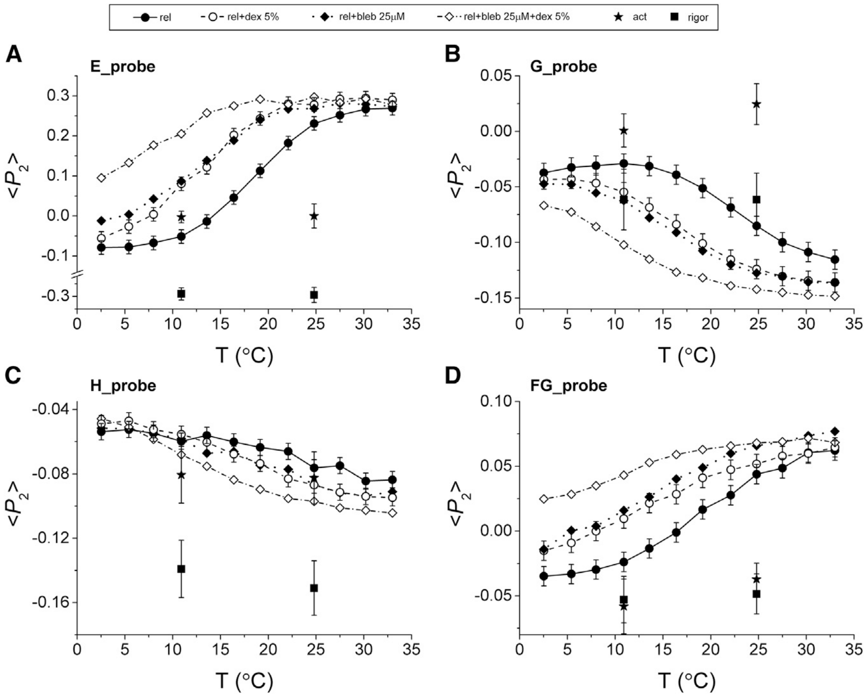

FIGURE 2 Temperature dependence of the $\langle P_2 \rangle$ order parameter for the RLC probes. Order parameter $\langle P_2 \rangle$ (mean ± pooled SD) of E (*A*), G (*B*), H (*C*), and FG (*D*) probes in the temperature range 2.5–33.0°C in standard relaxing solution (*solid circles* and *solid line*, $n = 3$ fibers), in the presence of 5% dextran T-500 (*open circles* and *dashed line*, $n = 3$ fibers) or 25 μM blebbistatin (*solid diamonds* and *dotted line*, $n = 1$ fiber), and in the presence of both 5% dextran and 25 μM blebbistatin (*open diamonds* and *dashed/dotted line*, $n = 1$ fiber). $\langle P_2 \rangle$ values during active contraction and in rigor at 11.0°C and 24.8°C are shown as stars ($n = 5$ fibers) and squares ($n = 3$ fibers), respectively (mean ± SD).

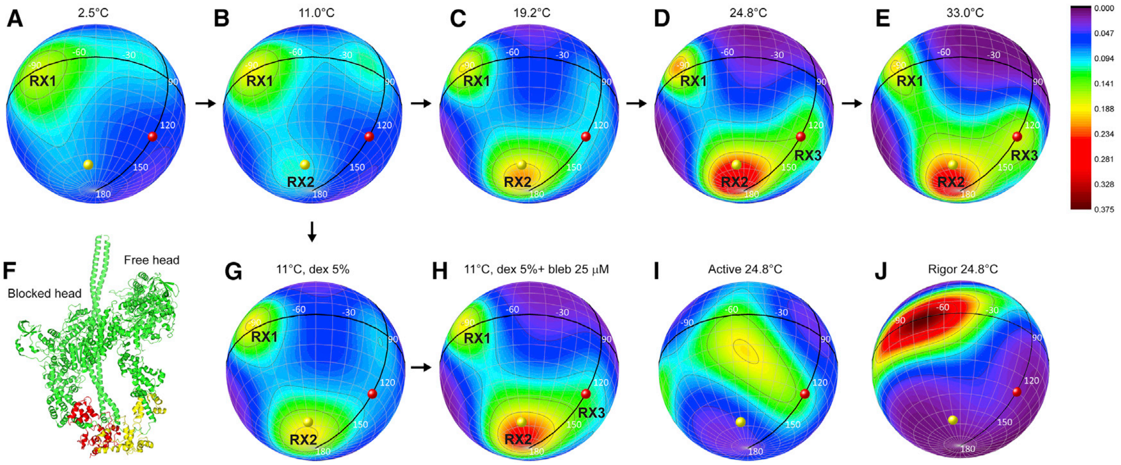

FIGURE 3 Orientation distributions of the RLC region of myosin. ME contour maps of the probability distribution of RLC orientations in skeletal muscle fibers shown as spherical plots, in which β is the latitude and γ is the longitude; $\beta = 90°$ at the equator and $\gamma = 0°$ at the meridian (*black lines*). (*A–E*) Standard relaxing solution in the temperature range 2.5–33.0°C, (*F*) IHM structure (3DTP (8)) with the RLCs of the blocked and free heads in red and yellow, respectively, (*G*) relaxing solution with 5% dextran at 11°C, (*H*) relaxing solution with 5% dextran + 25 μM blebbistatin at 11°C, (*I*) active contraction, and (*J*) rigor at 24.8°C. The RLC orientations for the blocked ($\beta = 131°$, $\gamma = 0°$), and free head ($\beta = 158°$, $\gamma = -60°$) in the EG frame are shown as red and yellow spheres, respectively, in the ME plots. The errors in the ME maps are analyzed in Fig. S9.

The in situ distribution of RLC orientations at 2.5°C in standard relaxing solution (in the absence of dextran and blebbistatin) showed a major equatorial population at $\beta = 90°$, $\gamma = -90°$ with ~20° angular dispersion in β and γ (RX1, Fig. 3 *A*). At 11°C two minor populations appeared, one close to the pole (RX2, $\beta = 165°$, $\gamma = -70°$) and one close to the equator ($\beta = 85°$, $\gamma = -15°$) (Fig. 3 *B*). RX2 increased progressively at higher temperatures with a consequent depletion of RX1, and became dominant above 19°C (Fig. 3, *C–E*; Movie S2). As the temperature was increased above 19°C, the minor equatorial population with γ near 0° became more parallel to the filament axis ($\beta = 130°$, $\gamma = 0°$, Fig. 3 *D*) and more intense (Fig. 3, *D* and *E*); this orientation is denoted RX3. Addition of 5% dextran at 11°C increased the intensity of RX2 and decreased that of RX1, so that the two peaks were roughly equal in intensity in these conditions (Fig. 3 *G*). Addition of 25 μM blebbistatin at 11°C increased the intensity of RX2 and RX3 (Fig. 3 *H*), so that the overall orientation distribution was similar to that in normal relaxing solution at 25°C (Fig. 3 *D*).

The hypothesis that the RX2 and RX3 peaks observed at higher temperatures in situ are similar to the conformation of the myosin heads in isolated thick filaments from tarantula muscle inferred from EM studies (3DTP (8), Fig. 3 *F*) was tested by calculating the (β, γ) orientation of the two RLCs in that structure. The orientations of the RLCs of the free and blocked heads of the IHM, shown as yellow ($\beta = 158°$, $\gamma = -60°$) and red ($\beta = 131°$, $\gamma = 0°$) spheres, respectively, in the spherical plots of Fig. 3, are similar to the orientation distribution of the two polar lobes (Table S2, Fig. S6). The RLC orientation of the free head (*yellow*) is close to RX2 ($\beta = 165°$, $\gamma = -70°$), whereas that of the blocked head is close to RX3 ($\beta = 130°$, $\gamma = 0°$), becoming more prominent at higher temperatures (Fig. 3 *E*).

The fraction of myosin heads with their RLC regions in either the RX2 or RX3 orientation ($F_{RX(2+3)}$) was estimated by integrating over the corresponding orientation region (see Fig. S8 for details) and normalizing by the total intensity at each temperature (Supporting Material). $F_{RX(2+3)}$ had a roughly sigmoidal dependence on temperature (Fig. 4, *solid circles*), which is similar to that of $\langle P_2 \rangle$ for the E helix probe (Fig. 2). At 2.5°C ~35% of the light chain domains are in the RX2 or RX3 orientation. $F_{RX(2+3)}$ increased with temperature and reached a maximum of ~70% at 25°C. At each temperature below 25°C the addition of dextran and/or blebbistatin induced an increase in $F_{RX(2+3)}$ (Fig. 4, *diamonds* and *open circles*), but did not significantly increase the limiting value of $F_{RX(2+3)}$ at 25°C and above.

Orientation distribution of the RLC region during active isometric contraction and in rigor

Fibers were calcium activated at 11.0°C or 24.8°C at fixed length using a temperature-jump protocol (21) in the absence of dextran. Active isometric force was 46 ± 4% (mean ± SE, $n = 17$) higher during activation at 24.8°C than at 11.0°C. In general, the order parameters during active contraction were distinct from those during relaxation (Figs. 2 and S4, *stars*). In the case of $\langle P_2 \rangle$ for the E helix probe the difference was small at 11.0°C, but about ~0.2 units at 24.8°C, indicating that the E helix, and therefore the long axis of the lever arm, becomes significantly less parallel to the filament axis on activation at this temperature.

The orientation distribution of the RLC region of the myosin heads during active isometric contraction at 11.0°C showed a broad peak centered on (β = 110°, $\gamma = -55°$) with ~±30° dispersion in β and γ, respectively (Fig. S7). The corresponding orientation distribution at 24.8°C was also broad, and centered on (β = 115°, $\gamma = -50°$) (Figs. 3 *I* and S7). The RX1, RX2, and RX3 orientations observed in relaxing conditions at 11.0°C and 24.8°C (Fig. 3, *B* and *D*) disappeared on activation.

In rigor conditions the order parameters of the E, FG, and H probes decreased from their relaxed values, and were insensitive to a temperature increase from 11.0°C to 24.8°C (Fig. 2, *squares*). $\langle P_2 \rangle$ for the E helix decreased by ~0.2 units at 11.0°C and by ~0.5 units at 24.8°C, indicating that the E helix axis and the myosin lever arm becomes much more perpendicular to the filament axis in rigor. The ME orientation distribution of the RLC region in rigor (Fig. 3 *J*) consisted of a single broad peak centered on ($\beta = 90°$, $\gamma = -80°$) with a larger dispersion in γ. The center of the rigor peak is close to the RX1 orientation seen in relaxing conditions.

DISCUSSION

Conformational equilibrium of myosin heads in relaxed muscle fibers

We used fluorescence polarization from four bifunctional probes on the C-lobe of the RLC to investigate the in situ conformation of the myosin heads in mammalian skeletal muscle. First, we characterized the conformational changes in the RLC region of the myosin heads during the temperature-induced structural transition in the thick filament in relaxing conditions (15–18). A temperature increase from 2.5°C to 33.0°C induced changes in the order parameters describing the orientation of each probe with respect to the filament or fiber axis (Fig. 2). The largest change was observed for the E helix probe dipole, which is almost parallel to the myosin lever arm: $\langle P_2 \rangle$ of the E helix increased by ~0.4 units as the temperature was increased, suggesting a large tilting of the probe, and therefore of the lever arm, toward an orientation more parallel to the filament. This is the first study, to our knowledge, in which such large temperature-induced changes of RLC orientation have been observed in relaxing conditions. A previous electron paramagnetic resonance spectroscopy study using RLC probes revealed temperature-dependent changes in the orientation and mobility of the probes in relaxing conditions, but did not provide a detailed description of the changes in RLC orientation (33). A previous fluorescence polarization study using bifunctional probes labeling the C-lobe of a smooth muscle isoform of RLC in skeletal muscle fibers showed very small effects of temperature in relaxing conditions (19). Here, we overcame some limitations of that study by using a skeletal muscle isoform of RLC rather than a smooth muscle isoform, and a milder RLC exchange protocol that replaces only ~25% of the native RLC. The mechanical properties of exchanged fibers were unaltered by this RLC exchange protocol (Fig. S2) and the large signals from the probes associated with temperature changes in relaxing conditions (Fig. 2) suggest that structure of the thick filaments was also preserved.

The results presented here reveal a temperature-dependent conformational equilibrium between the three main RLC orientations (RX1, RX2, and RX3) in relaxed muscle fibers. At 2.5°C, the most prominent RLC orientation has the E helix (and the myosin lever arm) roughly perpendicular to the filament axis (RX1, β = 90°, Fig. 3), similar to the orientation observed for actin-bound heads in rigor (Fig. 3 *J*). At 25°C, in contrast, the prominent orientations have the E helix much more parallel to the filament axis (RX2, β = 170° and RX3, β = 130°; Fig. 3). The RX2 and RX3 orientations are similar to those of the free and blocked heads, respectively, in the IHM observed in isolated thick filaments from invertebrate muscle (8). These results indicate that the majority of the myosin heads in relaxed muscle fibers at near-physiological temperature have their RLC regions in conformations similar to those in the IHM. The limitations of the technique presented here and the ME analysis, and of fitting crystal structures into EM electron densities, means that these conformations are not necessarily identical. Moreover, the results presented here indicate a higher fraction of RX2 (free-like) than RX3 (blocked-like) orientations at the higher temperatures, whereas these two fractions would by definition be equal

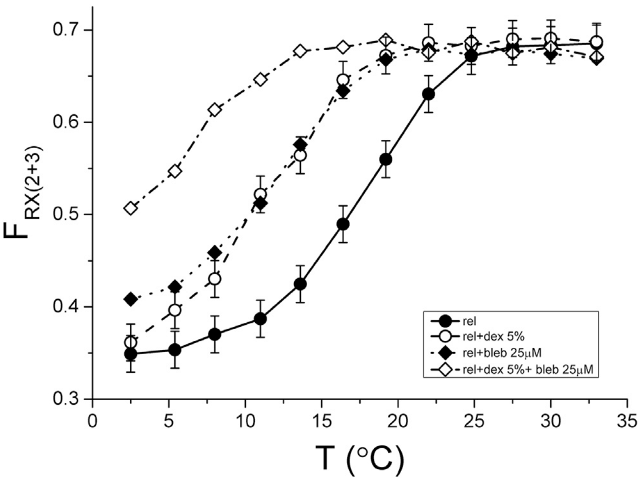

FIGURE 4 Temperature dependence of the fraction of myosin heads in the RX2 and RX3 orientations in relaxed muscle fibers. Fraction of myosin heads in the RX2 and RX3 orientations ($F_{RX(2+3)}$) calculated by integrating over RX2 and RX3 in the ME plots (Fig.3) under relaxing conditions (*solid circles* and *solid line*), in the presence of 5% dextran T-500 (*open circles* and *dashed line*) or 25 μM blebbistatin (*solid diamonds* and *dotted line*), and in the presence of both 5% dextran and 25 μM blebbistatin (*open diamonds* and *dashed/dotted line*). Errors are pooled SDs calculated as in Fig. 2.

for the IHM. Although ME analysis cannot give a precise measure of the relative probabilities of two closely-spaced orientations (28), the relatively large difference between RX2 and RX3 intensities suggests the presence in relaxed muscle fibers of some myosin molecules in a novel conformation, with one parallel (RX2- or free-like) and one perpendicular (RX1-like) light chain domain.

The sigmoidal temperature dependence of the fraction of parallel (RX2 or RX3) orientations (Fig. 4) is similar to that of the intensity of the first myosin layer line observed in x-ray studies (16). Moreover, the temperature-dependent increase in the fraction of RX2 or RX3 orientations is similar to that in the fraction of helically ordered heads estimated from the change in myosin layer-line intensities (17,34,35). Although the latter depend on other parameters of myosin head orientation, including their radial and azimuthal position and the conformation of the catalytic domain of the heads that cannot be detected by dipole probes on the RLC, there is a close correlation between a helically ordered conformation of the myosin heads and a parallel orientation of their light chain domains. Both of these sets of structural parameters are also correlated at least qualitatively with the formation of the IHM at higher temperature, which in turn is correlated with the increase in the fraction of heads in the so-called SRX state characterized by its slow ATP-turnover. The SRX state is estimated to be occupied by ~75% of the heads at physiological temperature in demembranated fibers from rabbit muscle (13,14), similar to the fraction of light chain domains in parallel (RX2 or RX3) orientations estimated here for similar conditions. Thus, the previous concept of a correlation between the IHM and SRX states is extended here by a direct correlation with the orientation of the RLC region of the myosin heads.

However, the results presented here also confirm previous evidence from relaxed muscle fibers at close to physiological temperature that ~1/3 of the myosin heads have their light chain regions in a more perpendicular conformation that is not consistent with the IHM. Modeling of x-ray data for these conditions suggested the coexistence of at least two myosin head orientations, corresponding to two radial positions with respect to the filament backbone (17), that are likely to be associated with different regions of the thick filament (36). EM-based three-dimensional reconstructions of the C-zone of vertebrate cardiac thick filaments showed two different conformations in the three axial layers of heads of each 43-nm repeat (10). Thick filaments from knockout mice lacking MyBP-C exhibit a more disordered structure (10), suggesting that some heads outside the C-zone may not form the IHM. Our results show that the RX1 myosin heads have a defined orientation of their light chain domains perpendicular to the filament axis, although these heads appear to be helically disordered in x-ray and EM studies (13,17,37). This distinction is likely to be related to the lack of sensitivity of the technique presented here to radial and azimuthal parameters, and to effects of changes in the conformation of the catalytic domains of the heads on the x-ray and EM data, as noted previously. The comparative advantage of the technique presented here is that it isolates the (β,γ) orientation of the RLC region of the myosin heads from those other parameters, and consequently shows that the helically disordered heads have RLC regions with ordered (β,γ) orientations. Our results show that the previous concept of the temperature-dependent change in myosin head conformation as a disorder-to-order transition (15–18) does not apply to the RLC region, which instead exhibits a temperature-dependent conformational equilibrium. The functional implications of this distinction are discussed below.

Factors that influence the conformational equilibrium of the RLC region of myosin

It has been shown that the formation of helical order in thick filaments requires the closed structure of the nucleotide-binding pocket of the myosin head (35,38). Therefore, the temperature dependence of the helical order has been explained on the basis that the open conformation is preferred at lower temperature, and the closed conformation at higher temperature (35). The open-closed equilibrium can also be influenced by ligands (32,35): in particular, the myosin inhibitor blebbistatin favors the closed conformation (20) and stabilizes the IHM in isolated myosin molecules (12), the helical order of the thick filament (32,39), and the SRX state of myosin (37). Our results show that blebbistatin also stabilizes the parallel orientations of RLC in relaxed muscle fibers by lowering the transition temperature of its conformational equilibrium without altering the maximum fraction of parallel RLC orientations at high temperature (Fig. 4).

We found that the filament lattice spacing also influences RLC orientation under relaxing conditions. The recovery of the filament lattice spacing typical of muscle cells with an intact surface membrane by adding 5% dextran T500 to relaxing solution (31) stabilized the parallel orientations of the RLC region in the relaxed demembranated fiber. The effect was similar to that of blebbistatin (Fig. 4). Moreover, the effects of blebbistatin and dextran on RLC orientation were additive, suggesting that both interventions alter the conformational equilibrium without altering the low- or high-temperature end states. In the presence of 5% dextran the center-to-center distance of thick and thin filaments decreases from 28 to 23 nm (31). Assuming a radius of 3.5 and 7.5 nm for thin and thick filaments, respectively, the distance between the filament surfaces decreased from 17 to 12 nm in the compressed lattice. Thus, either steric or charge effects may favor the parallel RLC orientation states if, as expected, these are associated with a lower mean radius of the myosin heads with respect to the thick filament axis. Alternatively, the effect of dextran on the

OFF structure of the thick filament might involve MyBP-C. The N-terminal region of MyBP-C is thought to bind to the thin filament, and therefore to be roughly perpendicular to the thick filament axis (40,41). These MyBP-C links have been shown to stabilize the resting structure of the thick filament by preserving its resting periodicity and the axial perturbations of the myosin layers in each 43-nm repeat (42). Therefore, the recovery of the physiological thick-thin filament distance by osmotic compression could promote the parallel RLC orientations by allowing the formation of MyBP-C links.

The experiments presented in this work were performed with unphosphorylated BSR-RLCs and the protocol used to prepare muscle samples yielded fibers in which the native RLC is unphosphorylated (13,43). However, under physiological conditions the conformational equilibrium of myosin could also be modulated by the phosphorylation level of RLC. It has been shown that under relaxing conditions RLC-phosphorylation can disorder the helical array of heads (13,44) and weaken the interactions between blocked and free heads (8). Therefore, RLC-phosphorylation is expected to disrupt the parallel orientation and shift the equilibrium toward the perpendicular orientation. This might be expected to bring the catalytic domains of the heads closer to the actin filaments and might therefore lead to changes in the kinetics of activation following electrical stimulation, as suggested by the finding that RLC-phosphorylation potentiates the twitch force in skeletal muscle (45).

Implications for myosin-based regulation of mammalian skeletal muscle

The results presented above indicate that under relaxing conditions at near-physiological temperature and lattice spacing, and at low RLC-phosphorylation levels, ~70% of the myosin heads have parallel RLC (RX2 and RX3) orientations, similar to those in the IHM/SRX state, with the myosin heads folded back onto the thick filament surface in a conformation that inhibits the myosin ATPase and the interaction with the overlapping thin filament. The remaining 30% of the heads have an RLC orientation (RX1) perpendicular to the filament axis and inconsistent with the IHM state. The 70% fraction of IHM-like heads appears to be an upper limit, because the addition of a stabilizing agent such as blebbistatin does not further increase either the fraction of heads with a parallel RLC region, as shown here, or the helical order (32).

Because the IHM seems to be confined to the C-zone of the thick filaments in vertebrate muscle (10,11), it is likely that the perpendicular RX1 orientation is confined to the remainder of the filament. The C-zone occupies ~1/3 of the filament, so the simplest interpretation of the distribution of RX1, RX2, and RX3 states observed here (Figs. 3 and 4), would be that all myosins in the C-zone in resting muscle are in the IHM state, but that the remaining two-thirds of the myosins outside the C-zone have, on average, one head with a parallel (RX2-like) RLC orientation and the other with a perpendicular (RX1-like) orientation.

The RX1, RX2, and RX3 orientations disappear on activation and are replaced by a broad orientation distribution lying between them (Figs. 3 *I* and S7). This transition is likely to be related to the complete loss of helical order of the heads on activation as measured by x-ray diffraction (3,6,42) and electron paramagnetic resonance (46). The redistribution of RLC orientations on activation of skeletal muscle does not occur on activation of cardiac muscle (26). This difference may be related to differences in the docking of the RLC onto the thick filament surface associated with the different isoforms of myosin and myosin binding protein C and with the different regulatory mechanisms and functional requirements in the two muscle types.

The signaling pathway that triggers the change in RLC orientation and the loss of helical order of the heads on activation of vertebrate skeletal muscle is unknown. In intrinsically regulated myosins this signal is provided by RLC phosphorylation, which weakens the interactions stabilizing the IHM and can be considered to switch myosin on. However, in skeletal muscle the structure of the thick filament becomes indistinguishable from the fully active state a few tens of milliseconds after the start of electrical stimulation (3). Because the skeletal isoform of myosin light chain kinase operates on a slower time scale (47), it cannot account for the fast activation of myosin. Thick filaments must therefore be switched on by a signaling pathway that rapidly transmits the on state of the thin filament to the thick filament. MyBP-C is a possible component of this signaling pathway, given its role in the stabilization of the resting structure of the thick filament and in the sensitization to calcium of the thin filament (48). MyBP-C links could sense the structural changes in the thin filament initiated by calcium binding to the regulatory proteins and trigger the exit of the heads from the IHM in the C-zone of the thick filament. Cooperative propagation of this structural change along the thick filament would be required to switch on all the myosin heads.

Although MyBP-C links may be involved in the regulation of the structural changes associated with myosin activation, our results suggest the possibility of an additional component involving the minor population of non-IHM heads in the resting thick filament. These heads might be more available for actin binding and could potentially act as sensors of the activation level of the thin filament (14). When the thin filament is switched on, allowing strong actin-myosin interactions, these perpendicular RX1 heads might contribute to disrupting the conformation of the IHM heads. Further work will be required to clarify the role of these different populations of myosin heads, of MyBP-C, and perhaps of titin, in thick filament regulation in vertebrate skeletal muscle.

CONCLUSIONS

In relaxed demembranated muscle fibers from mammalian skeletal muscle the RLC region of the myosin heads are in a temperature-dependent orientational equilibrium. A temperature increase from 2.5 to 33.0°C induces a shift of the conformational equilibrium from more perpendicular to more parallel orientations with respect to the thick filament axis. The latter are similar to the RLC orientations in the free and blocked heads of the IHM, and are stabilized by both blebbistatin and myofilament lattice compression. Close to physiological temperature and lattice spacing the heads with parallel RLC regions coexist with a significant fraction of myosin heads with a perpendicular orientation. During active contraction all these defined RLC orientations are replaced by a broad intermediate distribution. Modulation of the conformational equilibrium of the RLC region of the myosin heads may be a component of the physiological signaling pathway of muscle activation.

SUPPORTING MATERIAL

Supporting Discussion, nine figures, two tables, and two movies are available at http://www.biophysj.org/biophysj/supplemental/S0006-3495(15)00621-9.

AUTHOR CONTRIBUTION

L.F. and M.I. designed research, Z.H. contributed analytic tools, L.F. performed research and analyzed data, L.F. and M.I. wrote the article.

ACKNOWLEDGMENTS

The authors thank M. Rajaratnam (Randall Division of Cell and Molecular Biophysics, King's College London) for engineering support, A. C. Knowles (Randall Division of Cell and Molecular Biophysics, King's College London) for providing skeletal TnC, S. Perera and A. Jayo (Randall Division of Cell and Molecular Biophysics, King's College London) for their help with confocal microscopy.

This study was supported by the Wellcome Trust, UK. L.F. held a London Law Trust Medal Fellowship.

REFERENCES

1. Gordon, A. M., E. Homsher, and M. Regnier. 2000. Regulation of contraction in striated muscle. *Physiol. Rev.* 80:853–924.
2. Huxley, H. E. 1973. Muscular contraction and cell motility. *Nature.* 243:445–449.
3. Reconditi, M., E. Brunello, …, M. Irving. 2011. Motion of myosin head domains during activation and force development in skeletal muscle. *Proc. Natl. Acad. Sci. USA.* 108:7236–7240.
4. Fusi, L., E. Brunello, …, M. Irving. 2014. Structural dynamics of troponin during activation of skeletal muscle. *Proc. Natl. Acad. Sci. USA.* 111:4626–4631.
5. Somlyo, A. V., A. S. Khromov, …, A. P. Somlyo. 2004. Smooth muscle myosin: regulation and properties. *Philos. Trans. R. Soc. Lond. B Biol. Sci.* 359:1921–1930.
6. Huxley, H. E., and W. Brown. 1967. The low-angle x-ray diagram of vertebrate striated muscle and its behaviour during contraction and rigor. *J. Mol. Biol.* 30:383–434.
7. Woodhead, J. L., F. Q. Zhao, …, R. Padrón. 2005. Atomic model of a myosin filament in the relaxed state. *Nature.* 436:1195–1199.
8. Alamo, L., W. Wriggers, …, R. Padrón. 2008. Three-dimensional reconstruction of tarantula myosin filaments suggests how phosphorylation may regulate myosin activity. *J. Mol. Biol.* 384:780–797.
9. Wendt, T., D. Taylor, …, K. Taylor. 2001. Three-dimensional image reconstruction of dephosphorylated smooth muscle heavy meromyosin reveals asymmetry in the interaction between myosin heads and placement of subfragment 2. *Proc. Natl. Acad. Sci. USA.* 98:4361–4366.
10. Zoghbi, M. E., J. L. Woodhead, …, R. Craig. 2008. Three-dimensional structure of vertebrate cardiac muscle myosin filaments. *Proc. Natl. Acad. Sci. USA.* 105:2386–2390.
11. Al-Khayat, H. A., R. W. Kensler, …, E. P. Morris. 2013. Atomic model of the human cardiac muscle myosin filament. *Proc. Natl. Acad. Sci. USA.* 110:318–323.
12. Jung, H. S., S. Komatsu, …, R. Craig. 2008. Head-head and head-tail interaction: a general mechanism for switching off myosin II activity in cells. *Mol. Biol. Cell.* 19:3234–3242.
13. Stewart, M. A., K. Franks-Skiba, …, R. Cooke. 2010. Myosin ATP turnover rate is a mechanism involved in thermogenesis in resting skeletal muscle fibers. *Proc. Natl. Acad. Sci. USA.* 107:430–435.
14. Cooke, R. 2011. The role of the myosin ATPase activity in adaptive thermogenesis by skeletal muscle. *Biophys. Rev.* 3:33–45.
15. Wakabayashi, T., T. Akiba, …, Y. Amemiya. 1988. Temperature-induced change of thick filament and location of the functional sites of myosin. *Adv. Exp. Med. Biol.* 226:39–48.
16. Lowy, J., D. Popp, and A. A. Stewart. 1991. X-ray studies of order-disorder transitions in the myosin heads of skinned rabbit psoas muscles. *Biophys. J.* 60:812–824.
17. Malinchik, S., S. Xu, and L. C. Yu. 1997. Temperature-induced structural changes in the myosin thick filament of skinned rabbit psoas muscle. *Biophys. J.* 73:2304–2312.
18. Wray, J. 1987. Structure of relaxed myosin filaments in relation to nucleotide state in vertebrate skeletal muscle. *J. Muscle Res. Cell Motil.* 8:62a.
19. Brack, A. S., B. D. Brandmeier, …, M. Irving. 2004. Bifunctional rhodamine probes of Myosin regulatory light chain orientation in relaxed skeletal muscle fibers. *Biophys. J.* 86:2329–2341.
20. Kovács, M., J. Tóth, …, J. R. Sellers. 2004. Mechanism of blebbistatin inhibition of myosin II. *J. Biol. Chem.* 279:35557–35563.
21. Linari, M., M. Caremani, …, V. Lombardi. 2007. Stiffness and fraction of Myosin motors responsible for active force in permeabilized muscle fibers from rabbit psoas. *Biophys. J.* 92:2476–2490.
22. Romano, D., B. D. Brandmeier, …, M. Irving. 2012. Orientation of the N-terminal lobe of the myosin regulatory light chain in skeletal muscle fibers. *Biophys. J.* 102:1418–1426.
23. Ferguson, R. E., Y. B. Sun, …, M. Irving. 2003. In situ orientations of protein domains: troponin C in skeletal muscle fibers. *Mol. Cell.* 11:865–874.
24. He, Z. H., R. K. Chillingworth, …, M. A. Ferenczi. 1997. ATPase kinetics on activation of rabbit and frog permeabilized isometric muscle fibres: a real time phosphate assay. *J. Physiol.* 501:125–148.
25. Dale, R. E., S. C. Hopkins, …, Y. E. Goldman. 1999. Model-independent analysis of the orientation of fluorescent probes with restricted mobility in muscle fibers. *Biophys. J.* 76:1606–1618.
26. Kampourakis, T., Y. B. Sun, and M. Irving. 2015. Orientation of the N- and C-terminal lobes of the myosin regulatory light chain in cardiac muscle. *Biophys. J.* 108:304–314.
27. Rayment, I., W. R. Rypniewski, …, H. M. Holden. 1993. Three-dimensional structure of myosin subfragment-1: a molecular motor. *Science.* 261:50–58.

28. van der Heide, U. A., S. C. Hopkins, and Y. E. Goldman. 2000. A maximum entropy analysis of protein orientations using fluorescence polarization data from multiple probes. *Biophys. J.* 78:2138–2150.

29. Hopkins, S. C., C. Sabido-David, …, Y. E. Goldman. 2002. Orientation changes of the myosin light chain domain during filament sliding in active and rigor muscle. *J. Mol. Biol.* 318:1275–1291.

30. Sevrieva, I., A. C. Knowles, …, Y. B. Sun. 2014. Regulatory domain of troponin moves dynamically during activation of cardiac muscle. *J. Mol. Cell. Cardiol.* 75:181–187.

31. Kawai, M., J. S. Wray, and Y. Zhao. 1993. The effect of lattice spacing change on cross-bridge kinetics in chemically skinned rabbit psoas muscle fibers. I. Proportionality between the lattice spacing and the fiber width. *Biophys. J.* 64:187–196.

32. Xu, S., H. D. White, …, L. C. Yu. 2009. Stabilization of helical order in the thick filaments by blebbistatin: further evidence of coexisting multiple conformations of myosin. *Biophys. J.* 96:3673–3681.

33. Zhao, L., J. Gollub, and R. Cooke. 1996. Orientation of paramagnetic probes attached to gizzard regulatory light chain bound to myosin heads in rabbit skeletal muscle. *Biochemistry.* 35:10158–10165.

34. Xu, S., J. Gu, …, L. C. Yu. 1999. The M.ADP.Pi state is required for helical order in the thick filaments of skeletal muscle. *Biophys. J.* 77:2665–2676.

35. Xu, S., G. Offer, …, L. C. Yu. 2003. Temperature and ligand dependence of conformation and helical order in myosin filaments. *Biochemistry.* 42:390–401.

36. Oshima, K., Y. Sugimoto, …, K. Wakabayashi. 2012. Head-head interactions of resting myosin cross-bridges in intact frog skeletal muscles, revealed by synchrotron x-ray fiber diffraction. *PLoS One.* 7:e52421.

37. Wilson, C., N. Naber, …, R. Cooke. 2014. The myosin inhibitor blebbistatin stabilizes the super-relaxed state in skeletal muscle. *Biophys. J.* 107:1637–1646.

38. Zoghbi, M. E., J. L. Woodhead, …, R. Padrón. 2004. Helical order in tarantula thick filaments requires the "closed" conformation of the myosin head. *J. Mol. Biol.* 342:1223–1236.

39. Zhao, F. Q., R. Padrón, and R. Craig. 2008. Blebbistatin stabilizes the helical order of myosin filaments by promoting the switch 2 closed state. *Biophys. J.* 95:3322–3329.

40. Lee, K., S. P. Harris, …, R. Craig. 2015. Orientation of myosin binding protein C in the cardiac muscle sarcomere determined by domain-specific immuno-EM. *J. Mol. Biol.* 427:274–286.

41. Luther, P. K., H. Winkler, …, J. Liu. 2011. Direct visualization of myosin-binding protein C bridging myosin and actin filaments in intact muscle. *Proc. Natl. Acad. Sci. USA.* 108:11423–11428.

42. Reconditi, M., E. Brunello, …, G. Piazzesi. 2014. Sarcomere-length dependence of myosin filament structure in skeletal muscle fibres of the frog. *J. Physiol.* 592:1119–1137.

43. Szczesna, D., J. Zhao, …, J. D. Potter. 2002. Phosphorylation of the regulatory light chains of myosin affects Ca2+ sensitivity of skeletal muscle contraction. *J. Appl. Physiol.* 92:1661–1670.

44. Levine, R. J., R. W. Kensler, …, H. L. Sweeney. 1996. Myosin light chain phosphorylation affects the structure of rabbit skeletal muscle thick filaments. *Biophys. J.* 71:898–907.

45. Zhi, G., J. W. Ryder, …, J. T. Stull. 2005. Myosin light chain kinase and myosin phosphorylation effect frequency-dependent potentiation of skeletal muscle contraction. *Proc. Natl. Acad. Sci. USA.* 102:17519–17524.

46. Cooke, R., M. S. Crowder, and D. D. Thomas. 1982. Orientation of spin labels attached to cross-bridges in contracting muscle fibres. *Nature.* 300:776–778.

47. Kamm, K. E., and J. T. Stull. 2011. Signaling to myosin regulatory light chain in sarcomeres. *J. Biol. Chem.* 286:9941–9947.

48. Kampourakis, T., Z. Yan, …, M. Irving. 2014. Myosin binding protein-C activates thin filaments and inhibits thick filaments in heart muscle cells. *Proc. Natl. Acad. Sci. USA.* 111:18763–18768.

The conformation of myosin heads in relaxed skeletal muscle: implications for myosin-based regulation

Luca Fusi, Zhe Huang and Malcolm Irving

Randall Division of Cell and Molecular Biophysics, King's College London, London, SE1 1UL (UK)

<u>Supporting Material</u>

Text S1. Integration and Monte Carlo analysis of ME maps

When estimating the integral in spherical coordinates, the 2D-ME intensity distributions calculated at each temperature were multiplied by sinβ. A binary mask was used to select the lobe containing populations RX2 and RX3 (Fig. S8). The boundary of the mask was defined by the contour line of the ME map corresponding to a probability value of 0.045 and by a line running along the local minimum in the intensity between the RX1 and RX2 lobes. Each map was multiplied by the mask and the integral over the selected region was divided by the total over the whole map to calculate $F_{RX(2+3)}$, representing the fraction of heads with RLC regions in the RX2 or RX3 peak (Fig. 4).

In order to evaluate the error on the estimate of $F_{RX(2+3)}$ given the experimental errors on the order parameters, we applied a Monte Carlo simulation in which we calculated the ME maps in five iterations using simulated values of $\langle P_2 \rangle$ and $\langle P_4 \rangle$ for each probe, considered as normal random variables having mean and standard deviation as experimentally determined. The standard deviation (SD) of $F_{RX(2+3)}$ was calculated from the five values obtained in each iteration. This analysis was repeated for data at 2.5, 19.2, 33.0°C in the presence and in the absence of dextran (Fig. S9). The results of this analysis showed that both the relative intensity and position of the ME peaks are not significantly affected by small fluctuations in the order parameters of the four probes. The largest SDs of $F_{RX(2+3)}$ obtained were ±0.025 and ±0.015 for no-dextran and dextran respectively; the errors shown for each data point in Fig. 4 are the pooled standard deviation calculated from dextran and no dextran data.

Table S1. Angular coordinates of the C-lobe BSR-RLCs in the EG frame. θ is the angle between the dipole and the E helix axis, and φ is the angle between the plane defined by the E helix and the dipole and that defined by the E helix and G helix, all in the EG frame (see Fig. S1). L is the distance between the β-carbons of the two residues cross-linked by BSR.

Location	Mutation	L (Å)	θ	φ
E helix	D95CV103C	13.1	160.2°	-172.3°
G helix	E131CA138C	11.1	78.3°	-1.6°
H helix	K151CT158C	11.4	34.9°	-175.9°
Inter F-G helices	T122CK134C	11.9	61.2°	-56.8°

Table S2. Angular coordinates of relaxed RLC C-lobe orientations. β is the angle between the E-helix and the filament axis and γ is the rotation of the RLC around the E-helix.

	β	γ
Free RLC (3DTP)	158°	-60°
Blocked RLC (3DTP)	131°	0°
RX1	90°	-90°
RX2	165°	-70°
RX3	130°	0°

Supplementary Figures

Fig. S1

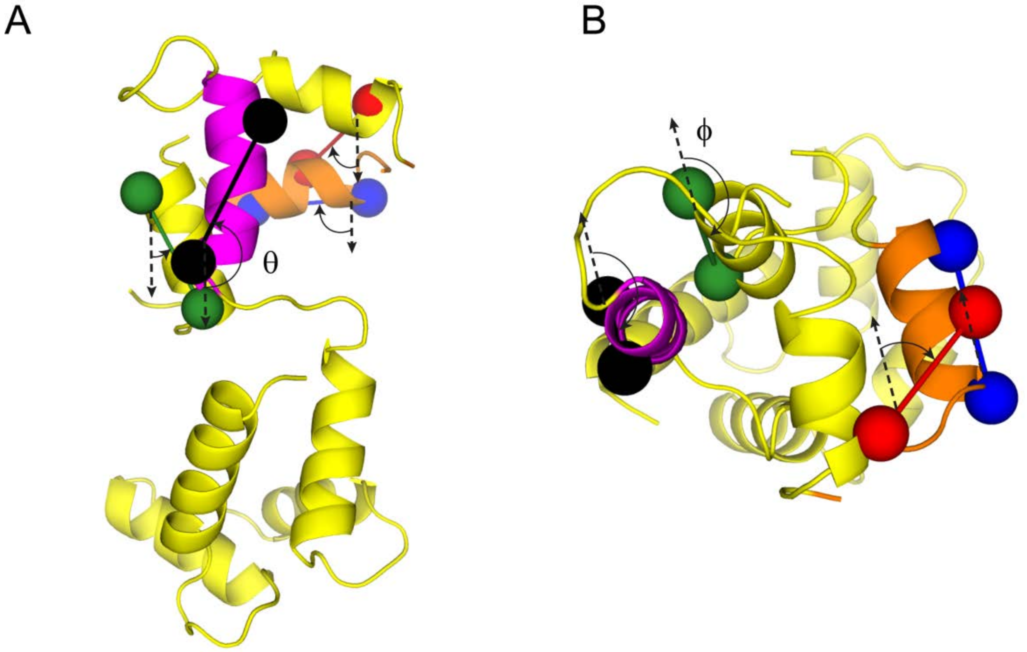

Representation of θ and φ angles of RLC probes in the EG frame. A) θ is the angle between the probe dipole and the vector parallel to the E helix axis (magenta). **B)** View of RLC C-lobe along the E helix axis showing φ, as the angle between the dipole axis and the vector parallel to the G-helix axis (orange). E helix probe, black; G helix probe, blue; H helix probe, green; FG probe, red.

Fig. S2

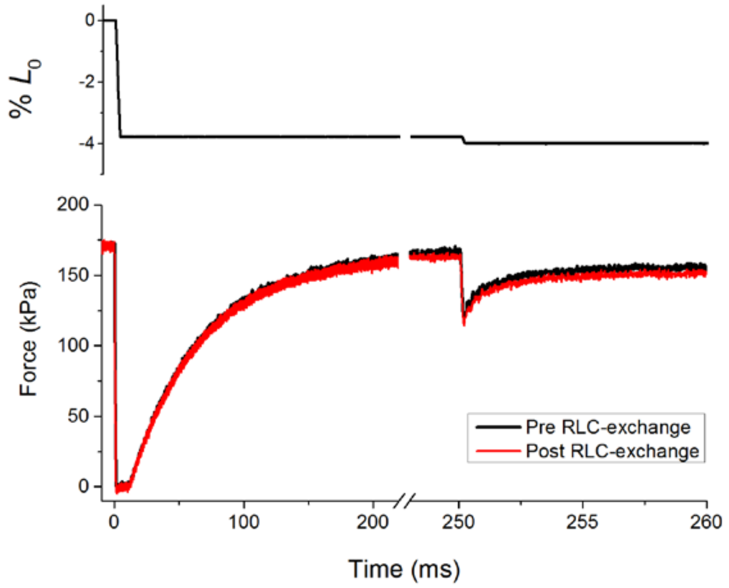

Effect of BSR-RLC exchange on fiber mechanics. Upper panel: imposed fiber length change. Ramp shortening (4% of the initial fiber length L_0 in 3 ms) was applied at the plateau of an isometric contraction; a step release (0.2% L_0 in 0.12 ms) was applied after force redeveloped to the isometric value. Lower panel: force response of the single muscle fiber before (black) and after (red) the BSR-RLC H-helix exchange measured by a capacitance force transducer. The force response to the step is shown on an expanded time scale.

Fig. S3

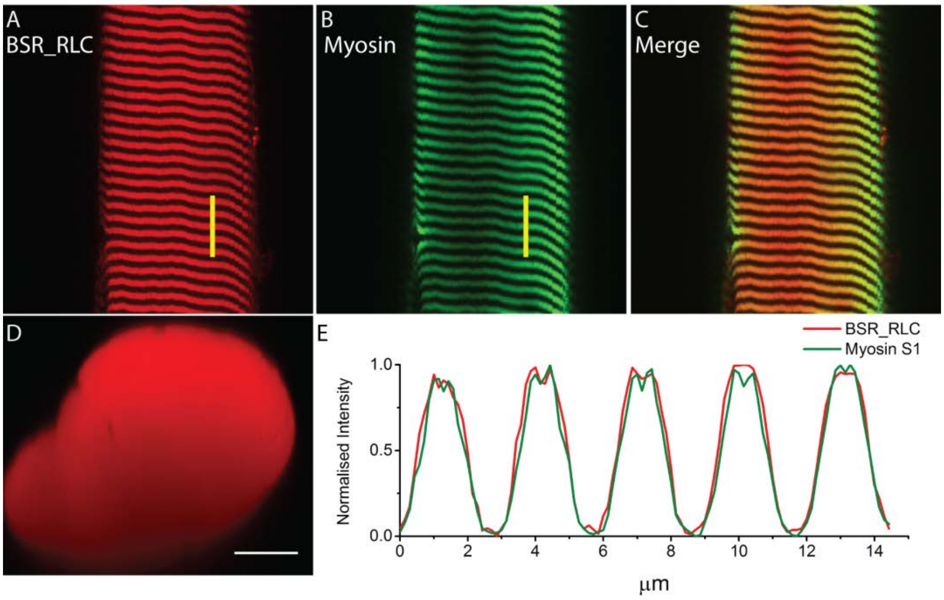

Confocal microscopy of BSR-RLC exchanged fibers. A) Fluorescence of an isolated muscle fiber exchanged with BSR-RLC (E-helix). B) Myosin staining with A4.1025 antibody. C) Merge of the two channels in A and B. D) Orthogonal view showing the fluorescence intensity distribution across the fiber cross-section. Scale bar, 15 µm. E) Normalized intensity profiles along the yellow bar in A and B.

Fig. S4

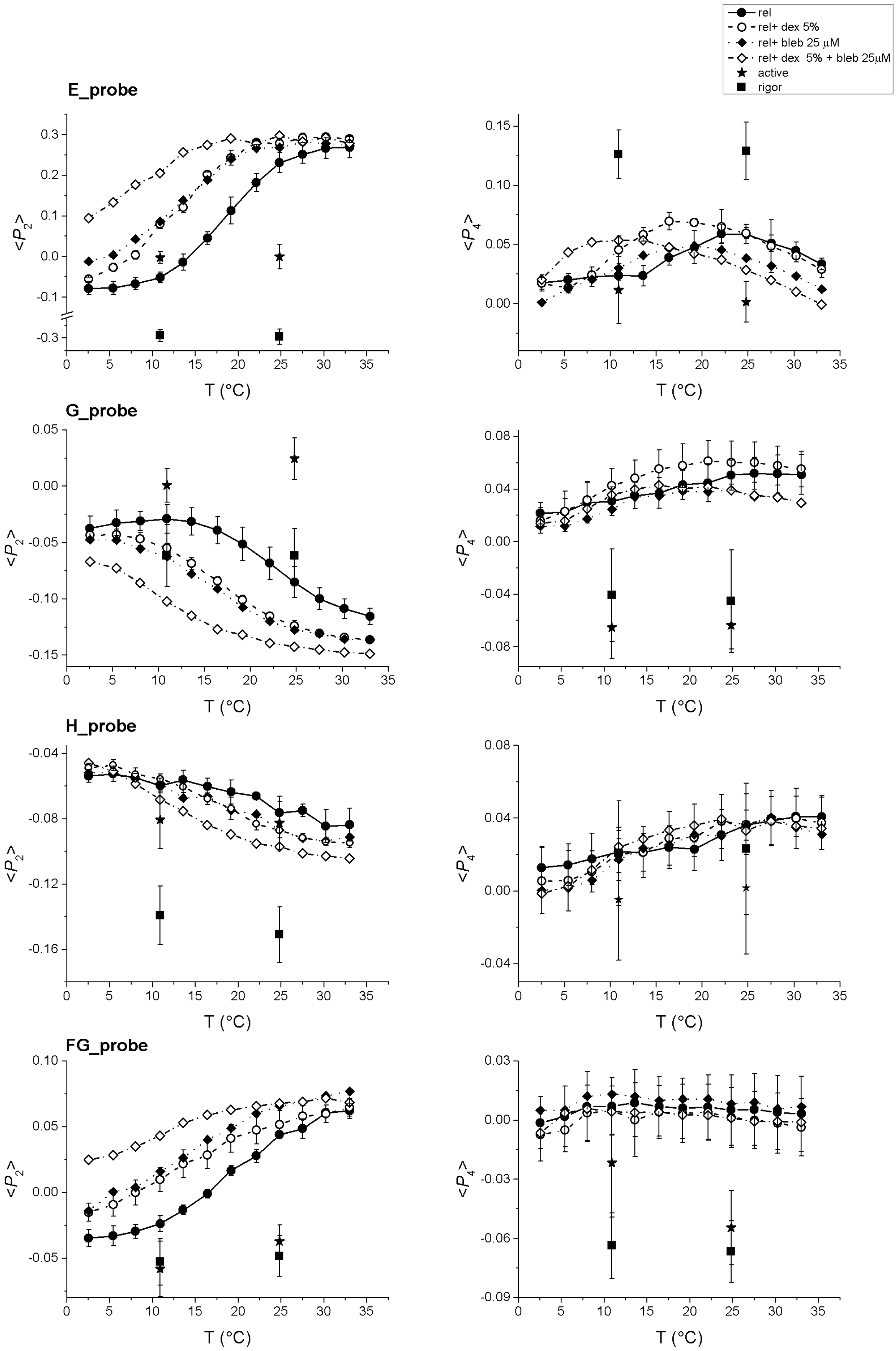

Temperature-dependence of <P_2> and <P_4> of the C-lobe BSR-RLCs. Order parameters <P_2> (left panels, same data as Fig.2 but without error pooling) and <P_4> (right panels)

(mean ± SD) of the four RLC probes in the temperature range 2.5-33.0°C in standard relaxing solution (filled circles and solid line, n=3 fibers), in the presence of 5% dextran T-500 (open circles and dashed line, n=3 fibers) or 25 µM blebbistatin (filled diamonds and dotted line, n=1 fiber), and in the presence of both 5% dextran and 25 µM blebbistatin (open diamonds and dashed/dotted line, n=1 fiber). Order parameters during active contraction and in rigor at 11.0°C and 24.8°C are shown as stars (n=5 fibers) and squares (n=3 fibres) respectively.

Fig. S5

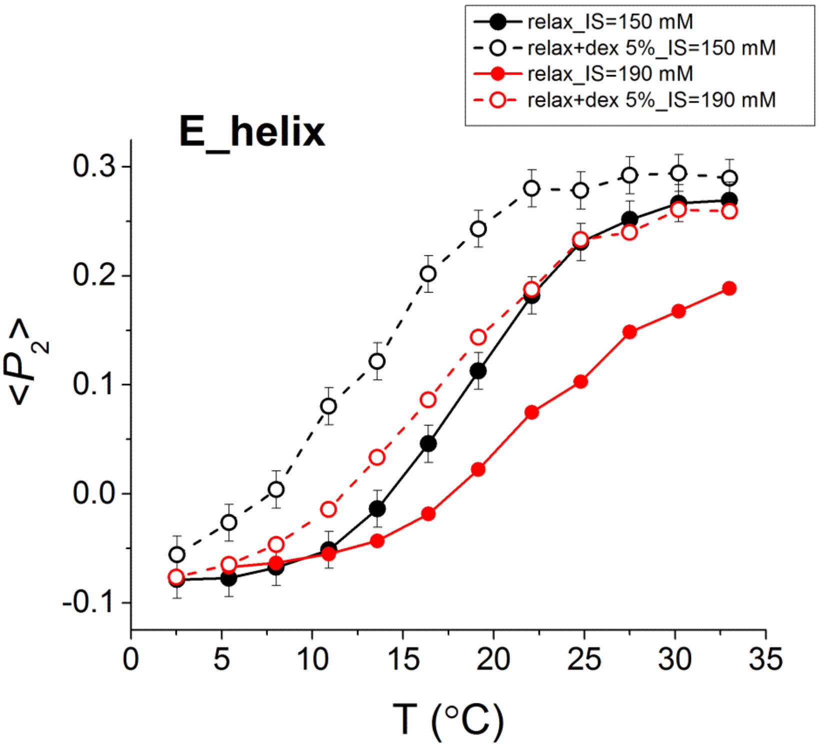

Effect of ionic strength on the orientation of the RLC E-helix probe. Temperature-dependence of the order parameter $\langle P_2 \rangle$ of the E-helix probe in the absence (filled symbols, solid line) and in the presence of 5% dextran T-500 (open symbols, dashed line) in relaxing solution with 150 mM ionic strength and composition given in Table 1 (black symbols, same as in Fig.2), and in relaxing solution with 190 mM ionic strength and with the following composition: TES 100mM, EGTA 25 mM, $MgCl_2$ 7.70 mM, Na_2ATP 5.5 mM, Na_2CP 20 mM, Glutathione 10 mM (red symbols).

Fig. S6

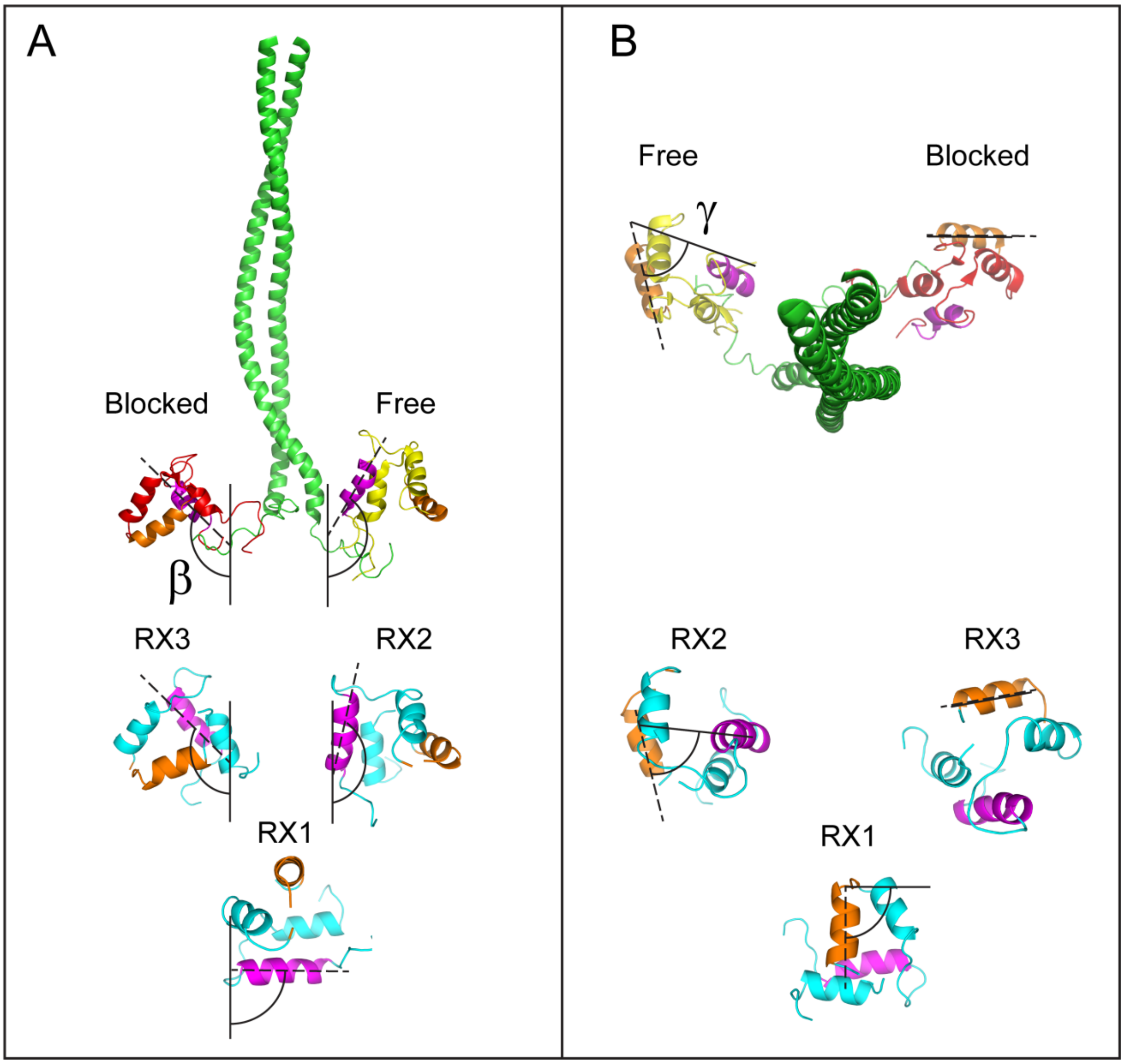

RLC C-lobe orientations in relaxed muscle. A) The angle β (see Table S2) between the E helix axis (magenta, dashed lines) and the filament axis (solid lines) is shown for the RLC C-lobes of the blocked and free heads in the IHM (3DTP), and for the *in situ* orientations RX1, RX2, and RX3. **B)** View along the filament axis showing γ, as the projected angle between the E helix axis (solid lines) and the G helix axis (orange, dashed lines), for each RLC C-lobe orientation.

Fig. S7

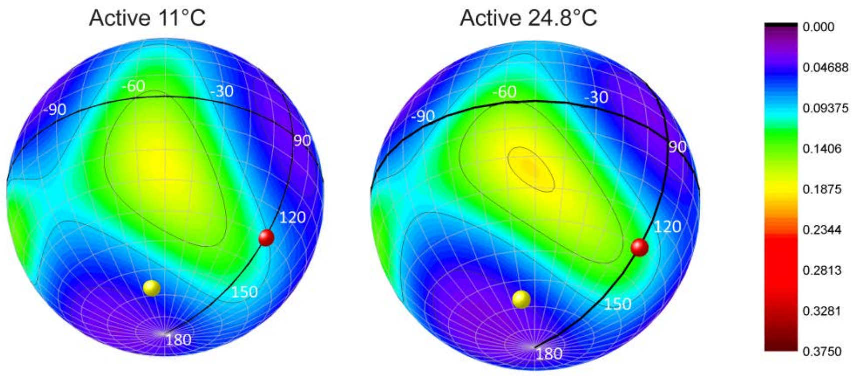

RLC orientation distribution during isometric contraction at 11.0°C and 24.8°C. Spherical plots of maximum entropy (ME) contour maps (as in Fig.3) showing the probability distribution of RLC orientations during active contraction. β=90° at the equator and γ=0°at the meridian. Red and yellow spheres correspond to the RLC orientations in the EG frame calculated for the blocked (β=131°, γ=0°) and free head (β=158°, γ=-60°) in the IHM (Fig. 3F).

Fig. S8

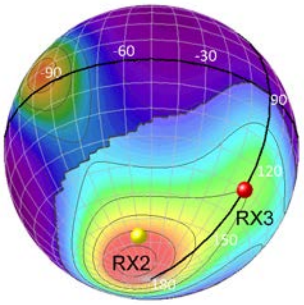

Integration of spherical ME plots. RLC orientation distribution at 24.8°C showing the integrated region of the map containing populations RX2 and RX3 (light colors).

Fig. S9

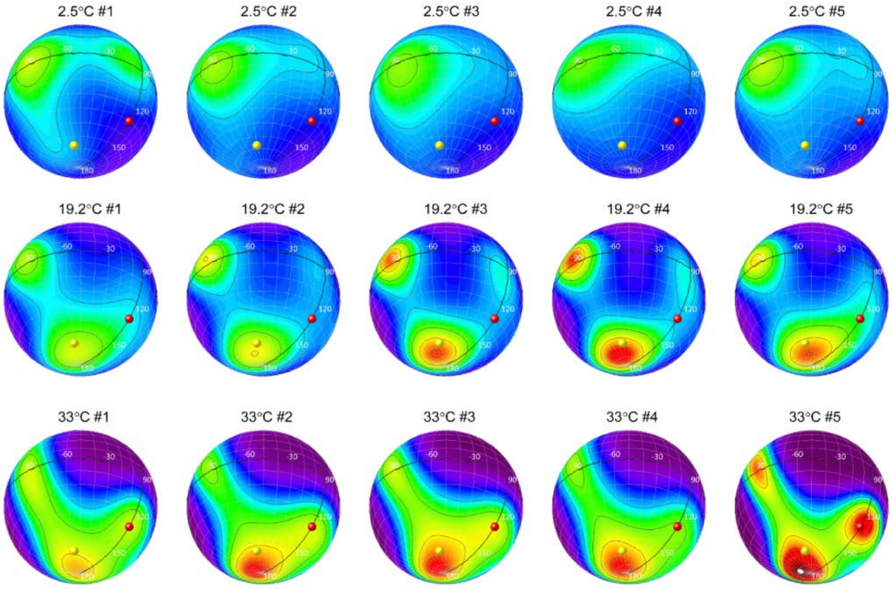

Monte-Carlo simulation of ME plots. RLC orientation distributions obtained in five iterations using simulated values of $<P_2>$ and $<P_4>$ for each probe at three temperatures (2.5, 19.2, 33°C).

Movie S1. Location of BSR probes in the C lobe of RLC. Bifunctional rhodamine (BSR) probes in the C-lobe of the regulatory light chain (RLC, yellow) are represented by rods cross-linking pairs of spheres denoting the inserted cysteines residues. E helix probe, black; G helix probe, blue; H helix probe, green; FG probe, red.

Movie S2. Temperature-dependence of RLC-orientation distribution in relaxing conditions. Frame-sequence of spherical plots of maximum entropy (ME) contour maps (as in Fig.3) showing the probability distribution of RLC orientations in the temperature range 2.5-33.0°C in standard relaxing solution. β=90° at the equator and γ=0°at the meridian. Red and yellow spheres correspond to the RLC orientations in the EG frame calculated for the blocked (β=131°, γ=0°) and free head (β=158°, γ=-60°) in the IHM (Fig. 3F).
